# Phylogenetics in a Warm Place: Computational Aspects of the Tropical Grassmannian

**DOI:** 10.1007/s11538-026-01773-6

**Published:** 2026-11-05

**Authors:** Samir Bhatt, John Sabol, Papri Dey, Matthew J. Penn, David Duchene, Ruriko Yoshida

**Affiliations:** 1 University of Copenhagen, Copenhagen, Denmark; 2 Imperial College London, London, UK; 3 Naval Postgraduate School, Monterey, California, USA; 4 University of California, Santa Cruz, USA; 5 Aalborg University, Aalborg, Denmark

**Keywords:** Phylogenetics, Minimum evolution, Tropical grassmanian, Tree space, Ultrametric, Tree metric

## Abstract

Phylogenetic trees provide a fundamental representation of evolutionary relationships, yet the combinatorial explosion of possible tree topologies renders inference computationally challenging. Classical approaches to characterizing tree space, such as the Billera–Holmes–Vogtmann (BHV) space, offer elegant geometric structure but suffer from statistical and computational limitations. An alternative perspective arises from tropical geometry: the tropical Grassmannian (${{\,\textrm{tropGr}\,}}(2,n)$), which Speyer and Sturmfels showed coincides with the space of phylogenetic trees. In this paper, we review the structure of the tropical Grassmannian and present algorithmic methods for its computational study, including procedures for sampling from the tropical Grassmannian. Our aim is to provide a computational perspective on these ideas for readers interested in phylogenetic tree space, and to motivate new research at the interface of tropical geometry and phylogenetic inference.

## Introduction

A phylogenetic tree is a mathematical structure that connects the worlds of evolutionary biology and computer science. In evolutionary biology, it represents the evolutionary relationships among a set of taxa. In computer science, an unrooted phylogenetic tree *T* on a set of n taxa is defined by a graph, i.e., T=(V,E), where *V* is the set of vertices and *E* the set of edges (often weighted). The tree *T* has exactly n leaves (also called tips), each corresponding to one taxon, and up to n-2 internal nodes, each of degree at least 3. When every internal node has degree exactly 3, *T* is called a *binary* tree. The number of binary trees on n taxa $\mathcal {T}_n$ is finite, but intractably large and given by the Schröder number (2n-5)!! in the unrooted case. For just n=40 individuals or species, the number of possible evolutionary trees is already greater than the number of hydrogen atoms in the Sun. Trying to find the correct tree from within that space is therefore a very challenging task. If the tree is rooted, the root node has out-degree 2, and there are n-1 internal nodes. In both rooted and unrooted cases, the edges *E* are typically assigned non-negative weights, representing evolutionary distances or divergence times.

The central challenge in computational phylogenetics is finding the best tree, $T^*$, given some data *X* and an objective criterion $\mathcal {L}$ that measures how well $T^*$ characterises the data, i.e.

1 $$\begin{aligned} T^* = \arg \min _{T \in \mathcal {T}} \, \mathcal {L}(X, T). \end{aligned}$$

Broadly three major objective criteria exist, maximum parsimony (Fitch 1971), maximum likelihood (Felsenstein 1983) and minimum evolution (Rzhetsky and Nei 1993). Solving Eq. 1 is NP-hard for all three criteria (Foulds and Graham 1982; Roch 2006; Fiorini and Joret 2012). This means that there is no known polynomial-time algorithm that always finds the globally optimal phylogenetic tree under standard objective criteria. Heuristic algorithms, such as hill climbing and tree rearrangement strategies often perform well in practice, but offer no guarantee of global optimality. Hill climbing performs well in phylogenetic tree search because the objective criteria landscapes on tree space exhibit strong local correlation, producing large basins of attraction around high-likelihood topologies and enabling local tree change moves to reliably ascend toward near-optimal trees. Therefore a central challenge in these heuristic algorithms is efficiently exploring tree space. This exploration is typically performed through local rearrangement operations that generate neighboring trees. Two common operations are Nearest Neighbor Interchange (NNI) and Subtree-Prune and Regraft (SPR). NNI modifies a tree by swapping subtrees across an internal edge, resulting in a new topology with minimal change. SPR involves cutting a subtree from the tree and reattaching it at a different edge, allowing for broader exploration of tree space. These operations define the neighborhood structure of the search, but naturally raise the question: what exactly is tree space, and how is it structured?

A geometrically rigorous solution to this question was provided by Billera et al. (2001), who showed that phylogenetic tree space can be endowed with the structure of a CAT(0)^1^ space, a type of non-positively curved metric space that admits unique geodesics between points. The Billera–Holmes–Vogtmann (BHV) tree space provides a geometric and combinatorial model for the space of phylogenetic trees with edge lengths. Formally, it is a piecewise Euclidean cubical complex in which each orthant (i.e., a Euclidean space ${\mathbb {R}}_{\ge 0}^k$) corresponds to a unique tree topology defined by a fixed set of internal splits. The dimension *k* of an orthant equals the number of internal edges in the tree, which is n-3 for a fully resolved unrooted binary tree with n leaves. Within each orthant, trees differ only in the lengths of their internal edges and are equipped with standard Euclidean geometry. Orthants are glued together along shared lower-dimensional faces corresponding to unresolved trees (i.e., trees with collapsed edges or polytomies). The resulting space is connected, contractible, and forms a CAT(0) space. This structure enables well-defined and efficiently computable geodesic distances between trees in polynomial time ($\mathcal {O}(n^4)$) (Owen and Provan 2011).

Despite its elegant geometric properties, BHV tree space presents certain challenges, particularly for statistical inference. One such issue is *stickiness*, a phenomenon arising from the non-manifold boundaries between orthants. Because the space is composed of orthants glued along lower-dimensional faces, geodesics and Fréchet means (i.e., the average tree) often lie on these lower-dimensional boundaries, corresponding to unresolved trees with one or more zero-length edges. As a result, even when input trees are fully resolved, their average under the BHV metric can be topologically unresolved. Additionally, the high-dimensional and combinatorial structure of BHV space makes some computational tasks, such as likelihood optimization or Bayesian posterior integration, difficult to implement efficiently, particularly as the number of taxa increases. In addition, Lin et al. (2017) showed that the dimension of the convex hull of three points in terms of the BHV metric over BHV space is unbounded in general, and that a convex hull in terms of the BHV metric over BHV space might not be closed. Therefore, unlike Euclidean space, it is not trivial to conduct a simple statistical analysis over BHV space. Finally, it is challenging to efficiently sample uniformly from the BHV space, again prohibiting Bayesian inference. These challenges have motivated the exploration of alternative models of tree space that better accommodate statistical and algorithmic needs.

A completely different way to fully characterise tree space that is still geometrically rigorous was provided by Speyer and Sturmfels (2004), which they termed the *Tropical Grassmannian*, and which serves a central role in our study. In what follows, we explore this exotically named object, motivating a description of tree space through the lens of tropical geometry, and deriving a few computational approaches along the way. Since our objective is to provide a practical introduction of the tropical Grassmannian that is accessible to evolutionary biologists and computational scientists, our treatment will tend to focus on intuition rather than formal proofs. Similarly, our algorithms are simple and easily implementable, offering researchers a means to immediately operationalize the concepts explored here and initiate their own explorations. We hope such efforts will spur further development of techniques from tropical geometry in tackling some of the significant challenges described earlier. Code for all figures are available at: https://github.com/bhattsamir/tropical_grassmannian.

An outline of our approach is as follows. We begin with a brief description of the structure of tree space and of equidistant tree space, followed by an introduction to the Grassmannian ${{\,\textrm{Gr}\,}}(2,n)$ and its tropical counterpart, the tropical Grassmannian ${{\,\textrm{tropGr}\,}}(2,n)$. We then motivate the derivation of a map that connects these two objects, and demonstrate how to pass from a point in ${{\,\textrm{Gr}\,}}(2,n)$ to ${{\,\textrm{tropGr}\,}}(2,n)$ via an algorithm that utilizes a 2-adic valuation. Leveraging the ultrametric structure of equidistant tree space, we then discuss methods for sampling from an ambient space along with efficient projection to equidistant tree space, an approach that explicitly leverages tropical convexity. We end with a demonstration of these sampling methods, along with some discussion regarding the distribution of tree topologies and application on an empirical dataset.

### The Structure of Tree Space

Before diving into tropical geometry, we review a few key concepts from the phylogenetic literature. We begin with a tree metric. A metric space $(X, {{\,\mathrm{\delta }\,}})$ (where *X* is the set of taxa of interest and ${{\,\mathrm{\delta }\,}}$ is some distance function) is called a *tree metric* if there exists a weighted tree *T* such that for all a,b∈X, ${{\,\mathrm{\delta }\,}}(a, b)$ equals the sum of edge weights along the unique path between *a* and *b* in *T*. Stated more simply, the leaf-to-leaf distance between any two taxa, is the sum of the branch lengths between them. Unfortunately, it is exceedingly unlikely that any estimated evolutionary distance matrices will meet this constraint. Therefore, one might ask, “is there a sufficient condition for a matrix to be tree metric?” Such a condition would then allow practitioners to know if a given distance matrix is valid. Given a tree, the vector of pairwise distances between taxa, typically taken as the lower-triangular part of the tree’s corresponding distance matrix, naturally defines a tree metric. However, for an arbitrary distance matrix (also called a dissimilarity map) to qualify as a metric, it must satisfy the following properties:

$$ \begin{aligned}&\text {(i) } {{\,\mathrm{\delta }\,}}(i,j) = 0 \iff i=j \qquad&\text {(Identity of Indiscernibles)}\\&\text {(ii) } {{\,\mathrm{\delta }\,}}(i,j) = {{\,\mathrm{\delta }\,}}(j,i) \quad \forall i,j&\text {(Symmetry)}\\&\text {(iii) } {{\,\mathrm{\delta }\,}}(i,j) \ge 0 \quad \forall i,j&\text {(Non-Negativity)}\\&\text {(iv) } {{\,\mathrm{\delta }\,}}(i,j) \le {{\,\mathrm{\delta }\,}}(i,k) + {{\,\mathrm{\delta }\,}}(k,j) \quad \forall i,j,k.&\text {(Triangle Inequality)} \end{aligned} $$

Further, for this distance matrix to be a tree metric, Buneman (1974) introduced a necessary and sufficient condition called the *Four-Point Condition*.

#### Theorem 1

(Four-Point Condition) Let $(X,{{\,\mathrm{\delta }\,}})$ be a metric space, and let $x_1,x_2,x_3,x_4 \in X$. Then

$$ {{\,\mathrm{\delta }\,}}(x_1,x_2) + {{\,\mathrm{\delta }\,}}(x_3,x_4) \,\le \, \max \bigl \{\, {{\,\mathrm{\delta }\,}}(x_1,x_3) + {{\,\mathrm{\delta }\,}}(x_2,x_4),\,\, {{\,\mathrm{\delta }\,}}(x_1,x_4) + {{\,\mathrm{\delta }\,}}(x_2,x_3) \bigr \}. $$

The theorem can be stated equivalently as the two largest sums among the following are equal:

2 $$\begin{aligned} {{\,\mathrm{\delta }\,}}(x_1, x_2) + {{\,\mathrm{\delta }\,}}(x_3, x_4), \quad {{\,\mathrm{\delta }\,}}(x_1, x_3) + {{\,\mathrm{\delta }\,}}(x_2, x_4), \quad {{\,\mathrm{\delta }\,}}(x_1, x_4) + {{\,\mathrm{\delta }\,}}(x_2, x_3). \end{aligned}$$

A proof of this theorem is available in most standard phylogenetic textbooks. However, it can be understood without a formal proof: in a tree, there is a unique path between any pair of nodes. When considering four leaves, the distances between them are constrained by the tree’s branching structure. Among the three possible ways to pair up the leaves into two disjoint pairs, the two pairs that share the longest common path through the tree will have the largest combined distances. The four-point condition captures this by requiring that the largest two of the three pairwise sums are equal. This reflects the fact that in a tree, any four points must fit into a consistent subtree structure, typically a quartet tree and the four-point condition detects whether such a structure exists.

The four-point condition characterizes tree metrics, meaning that if a distance matrix satisfies this condition, it is a tree metric and therefore represents a valid phylogenetic tree. Such a tree can then be efficiently reconstructed using algorithms such as neighbour joining (Saitou and Nei 1987). A sensible question would therefore be, as opposed to enumerating tree space by constructing discrete trees (Penn et al. 2024), is there a way to define the space of all possible trees through the four-point condition? We shall see later that this is exactly what the Tropical Grassmannian achieves.

An important observation regarding tree metrics is that they admit a natural decomposition. For a tree $$T\in \mathcal {T}_n$$ on n leaves, let *D* denote its associated n×n distance matrix, i.e., $$D(i,j)={{\,\mathrm{\delta }\,}}(i,j)$$. We assume^2^*T* is rooted, and identify this root with index 0. The height of a node $$h(i){:}{=}{{\,\mathrm{\delta }\,}}(0, i)$$ is its distance to the root. The edge connecting a leaf to its parent is its *pendant edge*. Adjusting node *i*’s pendant edge length by $$\Delta _i$$ modifies *D* according to $$D'(i,j)= D(i,j)+\Delta _i$$ applied to all j≠i. For any leaf with greatest height $$i^*\in \mathop {\textrm{argmax}}\limits _ih(i)$$, extending each pendant edge according to $$\Delta _i=h(i^*)-h(i)$$ results in a tree where every leaf has equal height. A rooted tree with this property is called an *equidistant tree*. An unrooted tree is equidistant if it admits a rooting that induces equal leaf heights. Thus, any tree *T* can be “extended” to an equidistant tree $T^{\prime }$ by adjusting pendant edge lengths. This yields

$$ D'(i,j) = D(i,j) + \Delta _i+\Delta _j={{\,\mathrm{\delta }\,}}_0(i,j)+2\,h(i^*)= 2\bigl (h(i^*)-(i\mid j)_0\bigr ) $$

where $${{\,\mathrm{\delta }\,}}_0(i,j){:}{=}{{\,\mathrm{\delta }\,}}(i,j)-{{\,\mathrm{\delta }\,}}(0,i)-{{\,\mathrm{\delta }\,}}(0,j)$$ denotes the standard Farris transform and $$(i\mid j)_0{:}{=}\tfrac{1}{2}[{{\,\mathrm{\delta }\,}}(0,i) + {{\,\mathrm{\delta }\,}}(0,j) - {{\,\mathrm{\delta }\,}}(i,j)]$$ denotes the Gromov product. Since for tree metrics, Gromov products measure the height of the *least common ancestor* ($$\textrm{lca}$$), i.e., $$(i\mid j)_0=h(\textrm{lca}(i,j))$$ given root 0, some authors use the vector of Gromov products (called the *cophenetic vector*) to represent a tree. The decomposition above shows the relationship between these various reparameterizations and provides some helpful intuition. Importantly, adjusting pendant edges does not affect internal edge lengths and therefore preserves the tree topology. Accordingly, $T^{\prime }$ captures the combinatorial structure of *T*, along with some additional terms to account for variations in pendant edge lengths.

#### Equidistant Trees and Ultrametric Spaces

Consider an equidistant tree *T*, and a triple of distinct leaves $\{i,j,k \}$. Since *T* is rooted, $\textrm{lca}(i,j,k)$ is well-defined as the unique internal node of maximal height lying on all three root paths from *i*, *j*, and *k*. Since *T* is equidistant, we have

$$ \delta (i,\textrm{lca}(i,j,k))=\delta (j,\textrm{lca}(i,j,k))=\delta (k,\textrm{lca}(i,j,k)). $$

Hence, $\textrm{lca}(i,j,k)$ induces a partition of the triple into a singleton,^3^ and a pair. Without loss of generality, assume *i* and *j* form the pair. Then

$$ \delta (i,j)\le \delta (i,k)=\delta (j,k)=2\cdot \delta (i,\textrm{lca}(i,j,k)). $$

This relation is called the *three-point condition*, and it characterizes equidistant trees.

##### Theorem 2

(Three-Point Condition) Let $(X,{{\,\mathrm{\delta }\,}})$ be a metric space, and let $x_1,x_2,x_3 \in X$. Then

$$ {{\,\mathrm{\delta }\,}}(x_1,x_2) \,\le \, \max \bigl \{\, {{\,\mathrm{\delta }\,}}(x_1,x_3),\,\, {{\,\mathrm{\delta }\,}}(x_2,x_3) \bigr \}. $$

The three-point condition implies a stronger form of the triangle inequality called the *ultrametric inequality*. For n leaves, the $$\left( {\begin{array}{c}n\\ 2\end{array}}\right) $$-dimensional space these inequalities describe is correspondingly called the *space of ultrametrics*, denoted as $\mathcal {U}_n$, and the elements therein, *ultrametrics*. Thus, any equidistant tree has an ultrametric distance vector, $d\in \mathcal {U}_n$. Intuitively, the three-point condition is equivalent to the statement: “the maximum of $\{{{\,\mathrm{\delta }\,}}(x_1,x_2), {{\,\mathrm{\delta }\,}}(x_1,x_3),{{\,\mathrm{\delta }\,}}(x_2,x_3) \}$ is achieved (at least) twice.” Geometrically, it implies that every triangle formed by ${{\,\mathrm{\delta }\,}}(i,j),{{\,\mathrm{\delta }\,}}(i,k),{{\,\mathrm{\delta }\,}}(j,k)$ is isosceles.

Ultrametric distance vectors induce a partition of the ground set (i.e., the leaves of *T*) analogous to the partition of leaf triples determined by their least common ancestor. This partition can be recovered by considering all triples and extracting the order relations on pairwise distances imposed by the ultrametric inequalities. In fact, this partition uniquely determines the topology of an equidistant tree: any two ultrametrics that induce the same set of inequalities yield the same tree topology. The collection of ultrametrics inducing a fixed topology is therefore defined by a system of linear inequalities and forms a cone in $$\mathbb {R}^{\left( {\begin{array}{c}n\\ 2\end{array}}\right) }$$. As the topology varies, the union of all such cones assembles into a fan.

We now see how the ultrametric component of a tree metric encodes the combinatorial structure of the tree. Thus, a method which efficiently samples from $\mathcal {U}_n$ can be used to sample from $\mathcal {T}_n$ by subsequently modifying the pendant edge lengths of a sampled ultrametric. In fact, sampling from $\mathcal {U}_n$ has a particular advantage over sampling from $\mathcal {T}_n$ directly. While neither $\mathcal {U}_n$ nor $\mathcal {T}_n$ are convex in the usual Euclidean sense, it turns out that the space of ultrametrics admits a weaker notion of convexity known as *tropical convexity*, which we define later. More specifically, $\mathcal {U}_n$ is a tropically linear space, a property which we will exploit in our computational methods.

#### The Grassmannian ${{\,\textrm{Gr}\,}}(2,n)$

The Grassmannian ${{\,\textrm{Gr}\,}}(r,n)$ is a widely used differentiable manifold. Here, we focus on the specific Grassmannian for r=2, i.e., ${{\,\textrm{Gr}\,}}(2,n)$, which is the space of 2-dimensional linear subspaces of an *n*-dimensional vector space defined over a field $\mathbb {K}$. In certain applications, it is desirable to use an algebraically closed field, such as the Puiseux series (a power series that allows fractional exponents, forming an algebraically closed field that ensures every polynomial equation has a solution). For ease of exposition here, however, we will identify $\mathbb {K}$ with the reals. Having done so, we can formally define:

3 $$\begin{aligned} {{\,\textrm{Gr}\,}}(2,n) = \left\{ V \subset {\mathbb {R}}^n \mid \dim (V) = 2 \right\} . \end{aligned}$$

Equivalently, we can define the Grassmannian as the span of all linearly independent pairs of *column* vectors:

4 $$\begin{aligned} {{\,\textrm{Gr}\,}}(2,n) = \left\{ \operatorname {span}(u, v) \mid u, v\in {\mathbb {R}}^n,\, \lambda _1,\lambda _2 \in {\mathbb {R}},\, \lambda _1 u+ \lambda _2 v= 0 \iff \lambda _1 = \lambda _2 = 0 \right\} . \end{aligned}$$

#### Plücker Coordinates and the Plücker Ideal

As we have noted, the Grassmannian ${{\,\textrm{Gr}\,}}(2,n)$ is the space of all 2-dimensional subspaces of ${\mathbb {R}}^n$ (or $\mathbb {K}^n$). To describe such subspaces, one can use a basis matrix (n×2 matrix), as in $$V:= \begin{pmatrix} u&v \end{pmatrix}$$, but this representation clearly isn’t unique since for any $\lambda _1,\lambda _2\ne 0$, $$V':= \begin{pmatrix}\lambda _1 u&\lambda _2 v\end{pmatrix}$$ span the exact same subspace. Plücker coordinates provide a more natural, invariant way to describe elements of ${{\,\textrm{Gr}\,}}(2,n)$.

The Plücker coordinates of a matrix *V* are given by the determinants of all maximal minors (the r×r submatrices) of *V*. Thus, given an ordering on the rows of *V* (to fix our indices), we can define the *Plücker vector* (ρ) as the image of a map that sends our matrix $V\in {\mathbb {R}}^{n\times r}$ to its unique embedding in projective space $\mathbb {P}^{(m-1)}$where $$m=\left( {\begin{array}{c}n\\ r\end{array}}\right) $$. In our case (r=2), we have:

5 $$\begin{aligned} &  V = \begin{pmatrix} u_1 & v_1 \\ u_2 & v_2 \\ \vdots & \vdots \\ u_n & v_n \end{pmatrix}, \quad {\rho }=\left( \,{\rho }_{ij}\mid 1\le i<j\le n, \right) , \quad \text {and}\nonumber \\ &  \quad {\rho }_{ij} := \det \begin{pmatrix} u_i & v_i \\ u_j & v_j \end{pmatrix} = u_i v_j - u_j v_i, \end{aligned}$$

Critically, all these Plücker coordinates *also* satisfy a series of quadratic Plücker relations given by the set of $$\left( {\begin{array}{c}n\\ 4\end{array}}\right) $$ quadrics of the form:

6 $$\begin{aligned} {\rho }_{ij} {\rho }_{kl} - {\rho }_{ik} {\rho }_{jl} + {\rho }_{il} {\rho }_{jk} = 0, \quad \text {for all } 1 \le i< j< k < l \le n. \end{aligned}$$

These quadratic Plücker relations are homogeneous in the coordinates ρ, which justifies viewing the Plücker vector projectively to ensure consistency under scaling.

The set of all such quadratic relations generates the Plücker ideal $\mathcal {I}(2,n)$ in the coordinate ring $\mathbb {K}[{\rho }]$. By construction, our Plücker vector resides in the common zero locus of these relations - namely, in the variety $\mathcal {V}$ defined by the Plücker ideal. That is, $\rho \in \mathcal {V}(\mathcal {I}(2,n))$.

Sampling from ${{\,\textrm{Gr}\,}}(2,n)$ is incredibly simple computationally, and is shown in Algorithm 1. Algorithm 1 performs a QR decomposition on an n×2 matrix with i.i.d. standard Gaussian entries, and then takes the column space of the orthonormalized matrix, yielding a random 2-plane in ${\mathbb {R}}^n$ that is distributed uniformly with respect to the Haar measure on ${{\,\textrm{Gr}\,}}(2,n)$. By subsequently applying the Plücker embedding to the output of Algorithm 1, the sampled 2-plane is represented by its Plücker coordinates as a well-defined point of $\mathbb {P}^{(m-1)}$, independent of the chosen orthonormal basis up to projective scaling.

**Figure:**
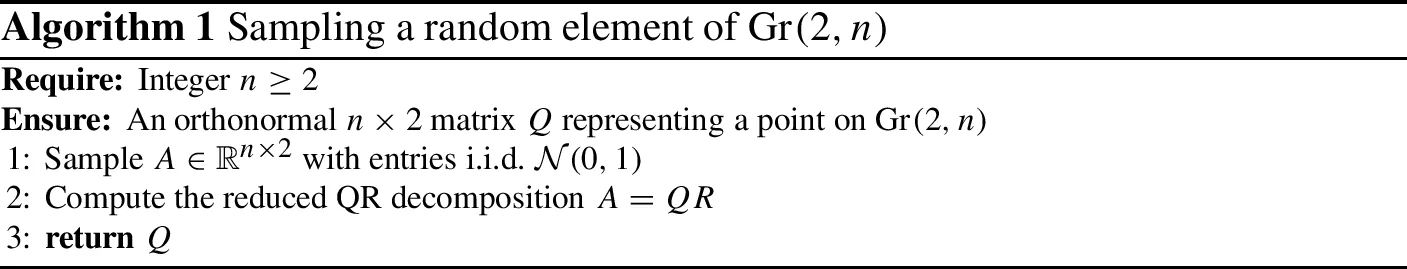

#### The Tropical Grassmannian

With a basic understanding of the Grassmannian ${{\,\textrm{Gr}\,}}(2,n)$, we now seek to transform it. In the seminal work of Speyer and Sturmfels (2004), the authors introduced a tropicalized version of the Grassmannian and prove this bijects to the space of phylogenetic trees and is isomorphic to the BHV space. To the unfamiliar reader, the tropical semiring (max-plus algebra), denoted as $\mathbb {T}_{\max }:=({\mathbb {R}}\cup \{-\infty \}, \oplus =\max , \otimes =+)$, has elements consisting of the real numbers and one additional value, -∞, to represent “zero.” In the tropical semiring the usual addition and multiplication operations are replaced by:

7 $$\begin{aligned} a \oplus b := \max (a, b), \quad a \otimes b := a + b, \end{aligned}$$

which explains why we refer to -∞ as the zero-element (-∞ is absorbing in max-plus multiplication). Operations over $\mathbb {T}_{\max }$ extend to naturally to vectors by considering component-wise application, thus yielding the tropical semimodule $\mathbb {T}_{\max }^n$.

The *tropical* Grassmannian, denoted ${{\,\textrm{tropGr}\,}}(2,n)$, is the *tropicalization* of ${{\,\textrm{Gr}\,}}(2,n)$. As the name implies, elements that live in $\operatorname {trop}{{\,\textrm{Gr}\,}}(2,n)$ operate via tropical addition and multiplication. Thus, the process of tropicalization implies a map $\textrm{val}:\mathbb {K}\rightarrow {\mathbb {R}}\cup \{-\infty \}$ such that the classical operations of multiplication and addition become their tropical analogues in the image space. This map $\textrm{val}$ is called a *valuation*, and it is central to our methods.

##### Definition 1

(A Field with Valuation) Consider a field $\mathbb {K}$, along with a map $\textrm{val}:{\mathbb {K}}\rightarrow {\mathbb {R}}\cup \{-\infty \}$ such that for any $a,b\in \mathbb {K}$ the following axioms hold:

8 $$\begin{aligned} \begin{aligned} \text {1. }&\textrm{val}(ab) = \textrm{val}(a) + \textrm{val}(b), \\ \text {2. }&\textrm{val}(a + b) \le \max \big (\textrm{val}(a), \textrm{val}(b)\big ), \\ \text {3. }&\textrm{val}(a) = -\infty \iff a=0. \end{aligned} \end{aligned}$$

Equipped with such a map, we say that $\mathbb {K}$ is a field with valuation, or a *valuated field*.

A valuation, loosely speaking, is a way to measure the “size” of elements in a field that respects multiplication and addition. At first glance, $\textrm{val}$ appears to operate just like a logarithm (log), for which the above axioms hold over $a,b\in \mathbb {R}_{\ge 0}$. Unfortunately, the restrictive domain of log disqualifies it from being a valuation in its own right, and an attempt at composition via log(|a|) (where |·| is the usual absolute value) fails the second axiom. Nonetheless, the intuition gained by thinking of valuations as a form of logarithmic mapping is the right one. Indeed, since in $\mathbb {T}_{\max }$ every element is greater than or equal to zero (i.e., -∞), the underlying set of our semiring is non-negative in a tropical sense. It turns out, that in order to satisfy the valuation axioms we need only to modify the absolute value from its usual definition (which satisfies the Archimedean property) to a *non-Archimedean* absolute value, which we denote using $|\cdot |_{\nu }$. That is, the relationship between a valuation map and its associated non-Archimedean absolute value is given by

$$ \textrm{val}(a){:}{=}\log (|a |_{\nu }) \quad \text {and} \quad |a |_{\nu } {:}{=}\exp \big (\textrm{val}(a)\big ), $$

and we sometimes say that the valuation *induces* the associated non-Archimedean absolute value. The following set of axioms follow directly.

##### Definition 2

*(Non-Archimedean Absolute Value)* For a field $\mathbb {K}$, we say that $|\cdot |_{\nu }$ defines a *non-Archimedean absolute value* on $\mathbb {K}$ if the following hold over any $a,b\in \mathbb {K}$:

9 $$\begin{aligned} \begin{aligned} \text {1. }\ &|ab |_{\nu } = |a |_{\nu }|b |_{\nu },\\ \text {2. }\ &|a+b |_{\nu } \le \max \big (|a |_{\nu },|b |_{\nu }\big ), \\ \text {3. }\ &|a |_{\nu } = 0 \ \Longleftrightarrow \ a=0. \end{aligned} \end{aligned}$$

Non-Archimedean absolute values make our earlier comment about valuations measuring the “size” of elements in a field ${\mathbb {K}}$ a bit more evident. Thus, we see that a valuated field ${\mathbb {K}}$ comes equipped with a non-Archimedean absolute value.

The choice of valuation (equivalently, the choice of non-Archimedean absolute value) and its computational realization is more important than it may initially appear. For example, what about $\textrm{val}(a)=0$ for all $a\ne -\infty$? All axioms are clearly met, though perhaps somewhat trivially. Indeed, this map can be applied to any field, and is called the *trivial valuation*. Such maps are not particularly interesting for our purposes, and so we will assume *non-trivial* valuations in what follows. An important special case is given by the field of rational numbers. For ${\mathbb {K}}={\mathbb {Q}}$, Ostrowski’s theorem states that every non-trivial absolute value is equivalent to either the usual real absolute value or to a *p-adic* absolute value for some prime *p*. Consequently, up to equivalence, the non-trivial non-Archimedean absolute values on ${\mathbb {Q}}$ are precisely the *p*-adic ones.

#### Max-Plus *p*-Adic Valuation

A *p*-adic valuation, well-known to number theorists, provides a classic example of a valuation map, and we use *p*-adic valuations in our tropicalization map that follows.

##### Definition 3

*(Max-Plus *p*-adic Valuation)* Let ${\mathbb {K}}:=\mathbb {Q}$ be the rationals. For a prime p and $x\in \mathbb {Q}^*$, write

$$ x=p^m\frac{a}{b}, \qquad a,b\in \mathbb {Z},\quad p\not \mid a,\ p\not \mid b. $$

Define the usual p-adic order by $\operatorname {ord}_p(x)=m$, and define the max-plus p-adic valuation by

$$ \operatorname {val}_p(x):=-\operatorname {ord}_p(x),\qquad \operatorname {val}_p(0):=-\infty . $$

The corresponding non-Archimedean absolute value is

$$ |x|_p:=p^{\operatorname {val}_p(x)}. $$

The p-adic absolute value on $\mathbb {Q}$ extends uniquely to its completion with respect to the metric induced by $|\cdot |_{p}$, called the field of *p*-adic numbers and denoted $\mathbb {Q}_p$. The associated p-adic valuation likewise extends uniquely to $\mathbb {Q}_p$. The field $\mathbb {Q}_p$ is an example of a non-Archimedean *local field*, i.e., a non-discrete locally compact topological field. Moreover, any non-Archimedean local field is either a finite extension of $\mathbb {Q}_p$ or a finite extension of $\mathbb {F}_p((t))$, the field of formal Laurent series over a finite field.

Readers familiar with the *p*-adics may wonder about the negative sign in the definition of $\textrm{val}_p(x)$, which distinguishes it from the usual definition of *p*-adic valuation. This negation is why we call this the “Max-Plus” *p*-adic valuation, and it is a consequence of our decision to operate using max-plus algebra (as opposed to the min-plus algebra where the *p*-adics typically operate). Loosely speaking, this exchange of semirings can be thought of exchanging the roles of the numerator and denominator for *x*. Switching from min-plus to max-plus (or vice versa) is a straightforward isomorphism given by $\min (a)=-\max (-a)$ where we remind the reader $\textrm{val}(-a)=\textrm{val}(a)$ applies to any valuation.

##### Example 1

For p=3, we have the following three-adic (max-plus) valuations and norms:

$$\begin{aligned} \textrm{val}_p(9)&= \textrm{val}_p(3^2) = -2,&\qquad |9 |_{p}&= p^{\textrm{val}_p(9)} = 3^{-2} = 1/9, \\ \textrm{val}_p\!\left( \tfrac{2}{3}\right)&= \textrm{val}_p(3^{-1}\cdot 2) = 1,&\qquad |\tfrac{2}{3} |_{p}&= p^{\textrm{val}_p(2/3)} = 3^1 = 3. \end{aligned}$$

Valuations can be applied in a vector space ${\mathbb {K}}^n$ in the natural way by applying $\textrm{val}$ component-wise to every element of a vector, that is $\textrm{val}({\textbf{x}})=\left( \textrm{val}(x_1),\ldots ,\textrm{val}(x_n)\right)$. Suppose we apply a valuation mapping to an arbitrary element of ${{\,\textrm{Gr}\,}}(2,n)$. By definition, this element satisfies the set of quadratic Plücker relations Eq. (6). Consider one such relation for any distinct indices i,j,k,l∈[n]: ${\rho }_{ij}{\rho }_{kl} - {\rho }_{ik}{\rho }_{jl} + {\rho }_{il}{\rho }_{jk} = 0$. Applying the valuation axioms reveals a tropical version of the Plücker relations called the *tropical Plücker relations*:

$$\begin{aligned}&\max \big ( \textrm{val}({\rho }_{ij}) + \textrm{val}({\rho }_{kl}),\, \textrm{val}({\rho }_{ik}) + \textrm{val}({\rho }_{jl}),\, \textrm{val}({\rho }_{il}) + \textrm{val}({\rho }_{jk}) \big ), \end{aligned}$$

is attained at least twice. Thus, any coordinate-wise valuation of a Plücker vector will satisfy a corresponding set of tropical Plücker relations. Since the tropical Plücker relations are equivalent to the four-point conditions in Theorem 1, we have an outline for a method that maps from a point in ${{\,\textrm{Gr}\,}}(2,n)$ to ${{\,\textrm{tropGr}\,}}(2,n)$.

Putting this all together yields the following (max-plus) tropicalization map τ via

10 $$\begin{aligned} \tau :\ {{\,\textrm{Gr}\,}}(2,n)({\mathbb {K}})\ \longrightarrow \ {{\,\textrm{tropGr}\,}}(2,n), \qquad {\rho }\mapsto \textrm{val}({\rho }), \end{aligned}$$

where $\operatorname {val}(\rho ) =\bigl (\operatorname {val}(\rho _{ij})\bigr )$ for $1\le i<j\le n$ is the component-wise valuation of the Plücker vector ρ.

A potential issue arises from this approach if the matrix *V* contains a singular 2×2 submatrix, i.e., ${\rho }_{ij}=0$. When this occurs, the resulting tropicalized coordinate becomes $\textrm{val}(\rho _{ij})=-\infty$, which violates our earlier assumption regarding the finiteness of tree metrics. We therefore modify our approach by mapping from the *open* Grassmannian

$$ {{\,\textrm{Gr}\,}}^{\circ }(2,n){:}{=}{{\,\textrm{Gr}\,}}(2,n) {\setminus } \bigcup _{i<j}\{{\rho }_{ij}=0\}, $$

and taking its projective closure after valuation. Thus, our tropicalization map works over the dense torus of the ambient projective space, which we now reiterate for clarity.

**Tropicalization Map.** Let $X=\mathcal {V}(\mathcal {I}(2,n))\subset \mathbb {P}^{m-1}$ denote the Plücker-embedded Grassmannian ${{\,\textrm{Gr}\,}}(2,n)$, where $$m=\left( {\begin{array}{c}n\\ 2\end{array}}\right) $$. To tropicalize X, one works on the dense torus of the ambient projective space,

$$ T^{m-1}:=\{[\rho _{ij}]\in \mathbb {P}^{m-1}:\rho _{ij}\ne 0 \text { for all } i<j\} \cong ({\mathbb {K}}^*)^m/{\mathbb {K}}^*, $$

where ${\mathbb {K}}^*={\mathbb {K}}{\setminus }\{0\}$. Accordingly, we set $X^\circ :=X\cap T^{m-1}$, so that all Plücker coordinates are nonzero and the valuation may be applied coordinate-wise. The Fundamental Theorem of Tropical Algebraic Geometry then states that

$$ \operatorname {trop}(X)=\overline{\operatorname {val}(X^\circ )}, $$

where the closure is taken in the Euclidean topology (equivalently, after projectivization, in $\mathbb {R}^m/\mathbb {R}\textbf{1}$, where $\textbf{1}$ is the all-ones vector in ${\mathbb {R}}^m$).

##### Remark 1

Our assumption regarding non-zero Plücker coordinates (i.e., applying the tropicalization map on the open Grassmannian) makes the implicit assumption that every pair of row vectors in *V* are linearly independent.

Thus, every pair of row vectors defines a basis in ${\mathbb {R}}^2$. By representing each row vector by its associated row index, the set E=[n] along with the independence relations as given by the collection of bases $\mathcal {B}$ defines a *matroid*^4^$M(E,\mathcal {B)}$. Here, we have the uniform matroid on *n* elements, $U_{2,n}$. To each basis $\{i,j\}$ of $U_{2,n}$, we assign a value as given by the valuation map applied to the coordinate $\rho _{ij}$ of the Plücker vector. This construction describes what Dress and Wenzel (1992) called a matroid *with valuation*, or a *valuated matroid*. The space of all such valuation vectors defines the *Dressian* of rank 2 on *n* elements, ${{\,\textrm{Dr}\,}}(2,n)$. In other words, ${{\,\textrm{Dr}\,}}(2,n)$ is equivalent to ${{\,\textrm{tropGr}\,}}(2,n)$, though this does not hold for r>2.

##### Example 2

Let n=6 and suppose V=(u,v) is a point in the open Grassmannian. A 2-adic tropicalization map produces the following tree, as shown in

$$\begin{aligned} V=\begin{pmatrix} 1 & 7 & 9 & 6 & 4 & 3\\ 9 & 3 & 2 & 5 & 7 & 4 \end{pmatrix}^{\top }, \quad \begin{aligned} {\rho }&=(-60,-79,-49,-29,-23,-13,17,37,19,33,55,30,22,9,-5)^{\top }\\ d&=\textrm{val}({\rho })=(-2,0,0,0,0,0,0,0,0,0,0,-1,-1,0,0)^{\top } \end{aligned} \end{aligned}$$

A few remarks regarding this tropicalization map are in order. First, note that the multiplicative homogeneity of the Plücker relations tropicalizes to additive homogeneity of the tropical Plücker relations. Concretely, if $V\in {\mathbb {K}}^{n\times 2}$ has columns u,v and we rescale them by $\lambda _1,\lambda _2\in {\mathbb {K}}^*$, i.e. $V'=(\lambda _1 u\,\,\,\lambda _2 v)=V\textrm{diag}(\lambda _1,\lambda _2)$, then every Plücker coordinate scales by the same factor: ${\rho }_{ij}'=\lambda _1\lambda _2\,{\rho }_{ij}$. Hence, for all i<j,

$$ \textrm{val}({\rho }_{ij}')=\textrm{val}({\rho }_{ij})+\textrm{val}(\lambda _1)+\textrm{val}(\lambda _2), $$

or equivalently,

$$ \textrm{val}({\rho }')=\textrm{val}({\rho })+{\zeta }\,\textbf{1}, \qquad {\zeta }:=\textrm{val}(\lambda _1)+\textrm{val}(\lambda _2). $$

This additive invariance explains our earlier statement regarding the closure of the tropicalization map occurring over $\mathbb {R}^{m}/\mathbb {R}\textbf{1}$, which is a quotient space called the *tropical projective torus*. Working in $\mathbb {R}^{m}/\mathbb {R}\textbf{1}$ means each vector $x\in \mathbb {R}^{m}/\mathbb {R}\textbf{1}$ is an equivalence class under the relation $x\sim x+{\zeta }\,\textbf{1}$. Practically, this means we may choose ζ so that $d:=\textrm{val}({\rho })+{\zeta }\textbf{1}$, thereby ensuring positive coordinates. Since adding ${\zeta }\textbf{1}$ preserves the tree-metric relations, sufficient translation ensures that *d* remains a valid tree metric, and has well-defined inter-leaf distances.

##### Remark 2

Given the translation invariance of $\mathbb {R}^{m}/\mathbb {R}\textbf{1}$, it is common practice to “normalize” such vectors in order to establish unique representatives. The *canonical coordinates*, for example, select the largest component-wise vector for *x* such that $x_i=0$ in at least one coordinate. Other common normalization schemes include enforcing $x_i=0$ for a *particular* *i*, or enforcing a mean-zero gauge such as $\sum _{i} x_i=0$.

**Lineality space of** ${{\,\textrm{tropGr}\,}}(2,n)$ **.** We see that ${\mathbb {R}}\textbf{1}\subseteq L_n$, where $L_n{:}{=}{{\,\textrm{lin}\,}}\left( {{\,\textrm{tropGr}\,}}(2,n)\right)$ and ${{\,\textrm{lin}\,}}(\cdot )$ denotes the lineality space.^5^ What about the rest of the lineality space? Suppose our parameterization of $V'=(\lambda _1 u\,\,\,\lambda _2 v)$ were modified so that rather than performing column scaling, we instead perform row scaling according to

$$\begin{aligned} V'=\begin{pmatrix} \mu _1u_1 & \mu _1v_1\\ \vdots & \vdots \\ \mu _nu_n & \mu _nv_n \end{pmatrix} . \end{aligned}$$

Following our tropicalization map as before, we have ${\rho }'_{ij}=\mu _i\mu _j{\rho }_{ij}$ so that $\textrm{val}({\rho }')_{ij}=\textrm{val}({\rho }_{ij})+\textrm{val}(\mu _j)+\textrm{val}(\mu _i)$. Unlike when we scaled column vectors, here the additive scaling is not uniform across the coordinates of $\textrm{val}({\rho }')$. That is, we have $\textrm{val}({\rho }')=\textrm{val}({\rho })+\textrm{val}(\mu )$ where $\mu :=(\mu _i\mu _j \mid 1\le i<j \le n)$. In this way, we can think of the $L_n$ as the containing the image of our tropicalization map when column *and* row scaling is performed on the input matrix *V*.

Are such row/column scaling sufficient in characterizing $L_n$? The answer, it turns out, is yes, and we now proceed a bit more formally, following (Maclagan and Sturmfels 2015), in order to properly define $L_n$.

The lineality space $L_n$ results from the homogeneity of the Plücker ideal $\mathcal {I}(2,n)$ with respect to the $\mathbb {Z}^m$-grading given by $\deg ({\rho }_{ij})=e_i+e_j \in \mathbb {Z}^m$. To see this, consider an arbitrary Plücker relation indexed by $\{i,j,k,l \}$. Then $\deg ({\rho }_{ij}{\rho }_{kl})=(e_i+e_j)+(e_k+e_l)=e_i+e_j+e_k+e_l$ is the same for each term in the relation. Thus, for the linear map

$$ \phi :\mathbb {R}^n \rightarrow \mathbb {R}^{\left( {\begin{array}{c}n\\ 2\end{array}}\right) }, \quad (u_1,\ldots ,u_n) \mapsto (u_i+u_j)_{1\le i<j\le n}, $$

we get that for any vector $\tilde{{\rho }}\in {{\,\textrm{tropGr}\,}}(2,n)$, if we set $\tilde{{\rho }}':=\tilde{{\rho }}+\phi (u)$, then ${\rho }'_{ij}={\rho }_{ij}+(u_i+u_j)$ in each coordinate entry. The corresponding *tropical* Plücker relations for $\tilde{{\rho }}'$ have terms that look like ${\rho }'_{ij}+{\rho }'_{kl}=({\rho }_{ij}+u_i+u_j)+({\rho }_{kl}+u_k+u_l)=({\rho }_{ij}+{\rho }_{kl})+(u_i+u_j+u_k+u_l)$. Clearly, all terms in the relation are shifted by the same additive constant so that the maxima remain unchanged. Since this is true of every relation, $\operatorname {Trop(X)}$ (the tropical variety) is invariant under translation by ϕ(u). In other words, $L_n$ is the image of the linear map ϕ, i.e.,

11 $$\begin{aligned} L_n={{\,\textrm{lin}\,}}({{\,\textrm{tropGr}\,}}(2,n))=\textrm{im}(\phi ) =span \Bigl \{\sum _{j\ne i} e_{ij}\ :\ 1\le i\le n\Bigr \} \subseteq {\mathbb {R}}^{\left( {\begin{array}{c}n\\ 2\end{array}}\right) }. \end{aligned}$$

Moreover, under the standard identification of ${{\,\textrm{tropGr}\,}}(2,n)$ with the space of tree metrics on [*n*], the lineality spaces agree:

$$ {{\,\textrm{lin}\,}}(\mathcal {T}_n)={{\,\textrm{lin}\,}}({{\,\textrm{tropGr}\,}}(2,n)), $$

where we remind the reader that $$\mathcal {T}_n\subseteq {\mathbb {R}}^{\left( {\begin{array}{c}n\\ 2\end{array}}\right) }$$ denotes the space of (phylogenetic) tree metrics on the leaf set $[n]=\{1,\dots ,n\}$, with coordinates indexed by pairs (*i*, *j*), $1\le i<j\le n$.

##### Remark 3

Examining Eq. (11), the vector $\sum _{j\ne i}e_{ij}$ (for a particular i∈[n]) has the effect of “extending/contracting” the pendant edge length of leaf *i* when considering the distance vector of the tree. That is, we can view the lineality space $L_n$ as the set of perturbations that adjust pendant edge lengths for the leaves of *T*.

An important special case arises when the second column of V has no zero entries. After rescaling row i by $v_i^{-1}$, we may replace V=(uv) by an equivalent matrix

$$ A= \begin{pmatrix} a_1 & a_2 & \ldots & a_n \\ 1 & 1 & \ldots & 1 \end{pmatrix}^{\top }, \qquad a_i=\frac{u_i}{v_i}. $$

For this representative, the Plücker coordinates are $\rho _{ij}=a_i-a_j$ for $1\le i<j\le n$. Thus the tropicalized Plücker vector has coordinates $d_{ij}:=\textrm{val}(\rho _{ij})=\textrm{val}(a_i-a_j)$. By the non-Archimedean valuation axiom, for any distinct i,j,k,

$$ d_{ik} = \textrm{val}(a_i-a_k) = \textrm{val}\bigl ((a_i-a_j)+(a_j-a_k)\bigr ) \le \max \{d_{ij},d_{jk}\}. $$

Applying the same argument cyclically shows that the maximum among $\{d_{ij},\, d_{ik},\, d_{jk}\}$ is attained at least twice. Since this applies to every triple $\{i,j,k\}$, it follows that $d=(d_{ij})$ satisfies the three-point condition for an equidistant tree.

If we interpret the row indices as nodes of a graph, and $d_{ij}$ as the edge weights between nodes *i* and *j*, then the three-point condition has the interpretation as a dependence relation between every triangle in the complete graph on *n* nodes, $K_n$. Hence, every triangle in the complete graph $K_n$, with edge weights $d_{ij}$, is isosceles in the ultrametric sense. These ultrametric conditions as applied to the triangles of $K_n$ immediately extend to *any* circuit of $K_n$. In this way, we can think of the space of ultrametrics $\mathcal {U}_n$ as a graphical matroid on the complete graph $M(K_n)$ whose dependence relations are given by the ultrametric condition applied to every circuit in the graph. We denote space whose coordinates obey these tropical dependence relations using $\operatorname {trop}(M(K_n))$. The correspondence between $\operatorname {trop}(M(K_n))$ and $\mathcal {U}_n$ was made explicit by Ardila (2005).

We are now ready to restate an important decomposition of our tropicalization map, which allows us to consider any tree (and therefore element of ${{\,\textrm{tropGr}\,}}(2,n)$) as the sum of an ultrametric and an element from the lineality space.

##### Lemma 3

((Maclagan and Sturmfels, 2015, Lemma 4.3.9)) Every tree metric $${\mathrm d}\in \mathbb {R}^{\left( {\begin{array}{c}n\\ 2\end{array}}\right) }/\mathbb {R}\textbf{1}$$ is an ultrametric $u\in \mathcal {U}_n$ plus a vector in the lineality space $L_n$. Thus, the space of phylogenetic trees has the decomposition

12 $$\begin{aligned} \mathcal {T}_n=\operatorname {trop}(M(K_n)) + L_n. \end{aligned}$$

We now have all the pieces to present a complete algorithm for sampling from the space of phylogenetic trees. The idea is simple: take any rational n×2 matrix *V* not possessing a singular 2×2 submatrix, compute its Plücker embedding ρ, apply a valuation component-wise to ρ, and translate the resulting vector as required to ensure positive pendant edge lengths. Here we utilize a 2-adic valuation. The procedure is provided in Algorithm 2. Since the length of the pendant edge incident to leaf *i* is given by

13 $$\begin{aligned} \ell _i=\frac{1}{2}\min _{\begin{array}{c} j,k\in [n]\setminus \{i\},\\ j\ne k \end{array}} \left( d_{ij}+d_{ik}-d_{jk}\right) , \end{aligned}$$

let $\ell _{\min }=\min _i\ell _i$. Translating each off-diagonal entry of the additive distance matrix by η increases every pendant edge length by η/2, while leaving all internal edge lengths unchanged. Consequently, translation by

$$ \eta =\max \{0, 2({\zeta }-\ell _{\min })\}, $$

where ${\zeta }>0$, produces an additive distance matrix whose pendant edge lengths are at least ζ.

**Figure:**
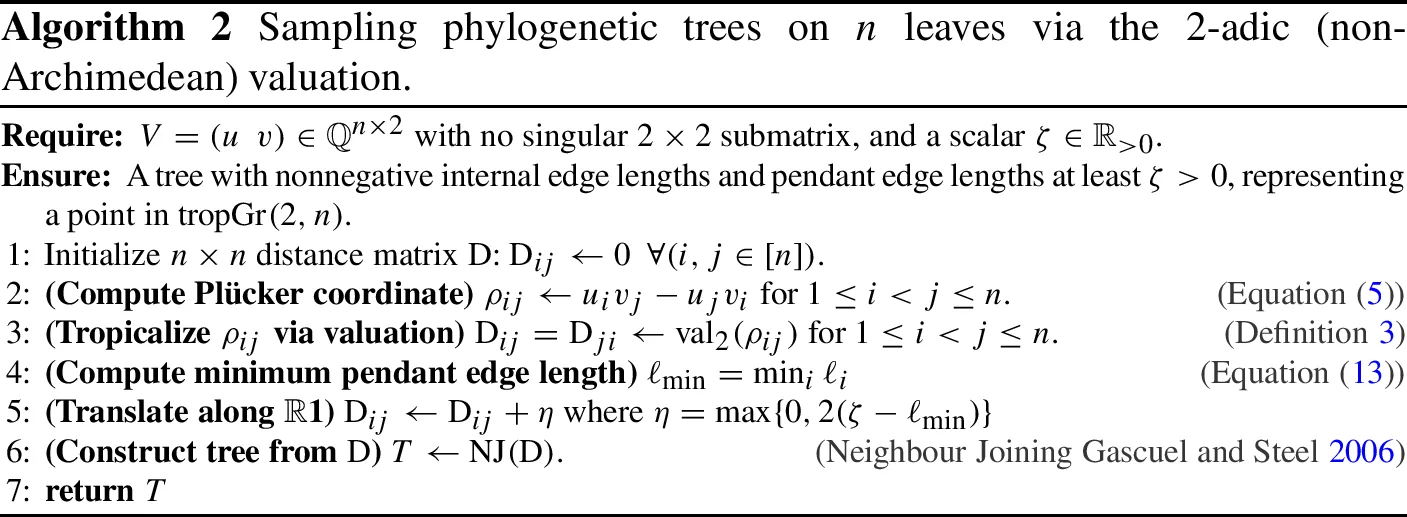

##### Remark 4

As outlined earlier, the aforementioned tropicalization map (and Algorithm 2) can be specialized by produce equidistant trees by forcing one column of *V* equal to $\textbf{1}$. Then, by the decomposition given in Lemma 3, one may sample each component separately and then add them in a manner that returns a vector in the positive orthant. Since the ultrametric portion determines the clade partitions, one can think of this component as fixing the tree’s topology while the lineality space adjusts particular branch lengths without modifying clade groupings.

##### Remark 5

For the theoretical definition of tropicalization, we take ${\mathbb {K}}$ to be an algebraically closed field equipped with a non-trivial valuation. For the explicit *p*-adic computations below, we work with rational points and the restriction of the *p*-adic valuation to $\mathbb {Q}\subset \overline{\mathbb {Q}}_p$. Such rational points produce valid points of the tropical Grassmannian, although they constitute only a discrete subset of the full real tropical variety.

##### Example 3

**Fig. 1:**
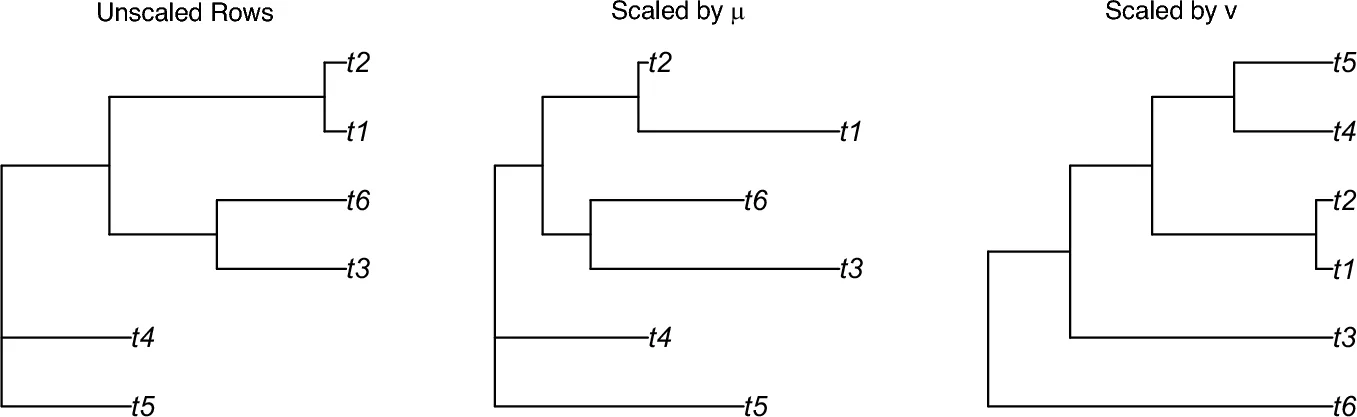
Trees resulting from the 2-adic tropicalization map. On the left, the map applied to the matrix *V* as given in Example 2. The center tree has rows of *V* scaled by μ as given in Example 3. The rightmost plot shows the result of scaling rows according by the second column vector (*v*), which results in an equidistant tree

Let *V* be as in Example 2 and μ=(3,4,1,2,7,2). Figure 1 shows the 2-adic tropicalization map applied *V* (left), $V^{\prime }$ whose rows are scaled by μ (center), and *A* given by the scaling *V* such that the second column equals $$\textbf{1}$$.

To summarize, a point in ${{\,\textrm{Gr}\,}}(2,n)$ may be represented by an n×2 matrix whose Plücker coordinate $\rho _{ij}$ is the determinant of the two rows indexed by *i* and *j*. Thus $\rho _{ij}$ records the linear dependence of this pair of row vectors. Under the max-plus 2-adic convention used here,

$$ \textrm{val}_2(\rho _{ij})=-\operatorname {ord}_2(\rho _{ij}), $$

so increasing divisibility of $\rho _{ij}$ by powers of 2 produces a *smaller* (more negative) valuation and a smaller 2-adic absolute value. In the normalized representation $A=(a\ \textbf{1})$, this is particularly transparent:

$$ d_{ij}=\textrm{val}_2(a_i-a_j). $$

If $a_i-a_j$ is divisible by a high power of 2, then $a_i$ and $a_j$ are close in the 2-adic metric and $d_{ij}$ is relatively small. After a common translation that produces nonnegative tree distances, such pairs remain relatively close and coalesce deeper in the corresponding equidistant tree. Thus the valuation converts *p*-adic proximity of the Plücker data into hierarchical proximity among leaves, producing a phylogenetic tree as the tropical image of the Grassmannian point.

##### Remark 6

(Distribution of 2-adic valuations of random differences and its effect on tree shape) Let $K\in \mathbb {N}$ and let u,v be independent and uniform on $\{0,1,\dots ,2^K\!-\!1\}$. Set $D\equiv u-v\pmod {2^K}$. Since subtraction is a bijection of the finite abelian group $\mathbb {Z}/2^K\mathbb {Z}$, the random variable *D* is uniform on $\mathbb {Z}/2^K\mathbb {Z}$. For 0≤k<K, the event $\textrm{val}_2(D)=k$ means that *D* is divisible by $2^k$ but not by $2^{k+1}$; these are exactly the residues $D=2^k(2m+1)$ with $0\le m<2^{K-k-1}$. Hence

$$ \mathbb {P}\bigl (\textrm{val}_2(D)=k\bigr )\,=\,\frac{2^{K-k-1}}{2^K}\,=\,2^{-(k+1)}, \qquad k=0,1,\dots ,K-1, $$

and $\mathbb {P}\bigl (\textrm{val}_2(D)\ge K\bigr )=2^{-K}$. Thus $\textrm{val}_2(D)$ is a geometric distribution truncated at *K*.

If instead *u*, *v* are sampled independently and uniformly from $\{1,\dots ,M\}$ with *M* large, then residue classes modulo $2^k$ are approximately equidistributed; in particular, for fixed *k* with $2^k\ll M$,

$$ \mathbb {P}\bigl (\textrm{val}_2(u-v)=k\bigr )\,\approx \,2^{-(k+1)}. $$

Consequently, for a *single* pair (*i*, *j*) of leaves, the marginal distribution of the 2-adic dissimilarity ${\mathrm d}_{ij}:=\textrm{val}_2(y_i-y_j)$ is (approximately) geometric. Across many pairs these values are *not* independent, but there is still a strong tendency for $d_{ij}$ to take only a few small integer values. This creates large blocks of exact ties among quartet sums in the four-point test, which *on average* promotes more balanced topologies (many clades coalescing at the same level).

It is important to note that when we say that pairwise distances are geometric, this refers only to the marginal distribution of $\textrm{val}_2(y_i-y_j)$ for a fixed pair. These values are not independent, and must satisfy the constraints of a tree metric.

Tropicalization and geometric distributions over local fields are closely related. In Tran (2020), Tran proves that the image of the one-dimensional *p*-adic Gaussian under the valuation map is geometric, and observes that this result follows immediately from a theorem of Evans (2001). The truncated geometric law satisfied by $\textrm{val}_2(D)$ observed here may therefore be viewed as a finite-level analogue of Tran’s result, arising from the geometric decay in the number of residue classes divisible by increasing powers of 2.

##### Example 4

*(*2*-adic ladder (caterpillar) from powers of *2*)* Let $y_i=2^{\,i-1}$ for i=1,⋯,n and define ${\mathrm d}_{ij}:=\textrm{val}_2(y_j-y_i)$ for i<j. Then

$$ y_j-y_i \,=\, 2^{i-1}\bigl (2^{\,j-i}-1\bigr ) $$

so $\textrm{val}_2(y_j-y_i)=i-1$. In particular,

$$ {\mathrm d}_{12}=0,\quad {\mathrm d}_{23}=1,\quad {\mathrm d}_{34}=2,\,\dots ,\, {\mathrm d}_{(n-1)n}=n-2, $$

and more generally ${\mathrm d}_{ij}=i-1$ for every i<j. The 2-adic ultrametric inequality $\textrm{val}_2(x-z)\le \max \{\textrm{val}_2(x-y),\textrm{val}_2(y-z)\}$ is satisfied, and the hierarchical pattern is strictly nested: the pair $\{n-1,n\}$ coalesces deepest, then $\{n-2,\{n-1,n\}\}$, and so on. The resulting rooted tree is a maximally imbalanced *caterpillar* (a single backbone with leaves attaching one by one at increasing depth).

**Fig. 2:**
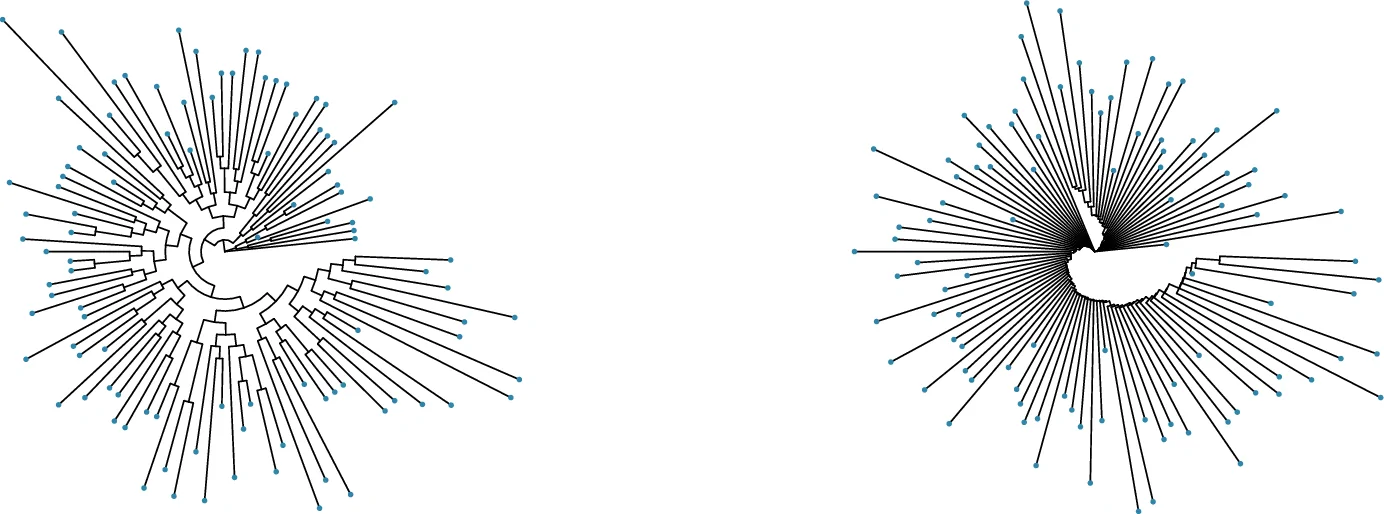
Two phylogenetic trees with n=100 leaves obtained using the same n×2 matrix *V*. The tree on the left is obtained by tropicalizing the Plücker vector using a 2-adic valuation in the manner of Algorithm 2. The tree on the right is obtained using a tropical Stiefel map

P-adic valuations, while conceptually simple, present non-trivial computational challenges, and working over such fields can be intractable for many real-world applications. At this point, one might wonder about the Plücker embedding itself and whether, by computing determinants tropically, one can attain a tropical Plücker vector directly from a n×2 matrix *V* rather than tropicalizing (by applying coordinatewise valuations) the Plücker vector attained from the usual embedding. If we define $\tilde{{\rho }}_{ij}:=u_i\otimes v_j \oplus u_j\otimes v_i$ we see that the resulting vector $\tilde{{\rho }}$ satisfies the tropical Plücker relations by construction, and thus also satisfies the four-point condition. Such a construction is called the *tropical Stiefel map* (Fink and Rincón 2015). Then by appropriate translation into the positive orthant as before we arrive at a tree metric. Unfortunately, the authors of Fink and Rincón (2015) show that this map is only capable of producing caterpillar trees and as such, provides little utility for our purposes here. Figure 2 provides a comparison of the trees produced using the method of Algorithm 2 (left) and the tropical Stiefel map (right).

## Sampling over ${{\,\textrm{tropGr}\,}}(2,n)$

Numerous methods exist for sampling from tree space, yet vanishingly few can generate samples that are uniformly distributed across the entire space. A phylogenetic tree constructed from an input alignment often exhibits substantial variability and uncertainty. Therefore, rather than focusing on a single estimated tree, it is natural to consider probability distributions over the space of phylogenetic trees for statistical analysis. For example, Garba et al. developed a new space of phylogenetic trees, called the Wald Space, in which well-defined probability distributions can be used for statistical inference on trees (Garba et al. 2019). Among such distributions, the uniform distribution is fundamental, since it provides a natural baseline measure on tree space and can serve as a building block for sampling from more complex distributions. In Euclidean settings, for instance, simple reference distributions can be transformed to generate samples from other distributions, as in the Box–Muller method for producing Gaussian random variables. Similarly, identifying a uniform distribution on phylogenetic tree space is a useful first step toward probabilistic and statistical analysis in that setting. Thus, determining a uniform distribution is useful not only as a baseline for statistical analysis, but also as a way to better understand the geometric structure of phylogenetic tree space.

Tree space is typically characterised as ranging between two structural extremes: the star tree and the caterpillar (ladder) tree. Perhaps the simplest approach is the Proportional to Distinguishable Arrangements (PDA) model, which places the uniform distribution on the space of all combinatorially distinct tree topologies with labelled leaves. Representing trees via a bijection to integer vectors (e.g. Penn et al. 2024), one can equivalently view PDA as uniform sampling in that vector space. Maximum-likelihood tree inference (Minh 2020) does not explicitly define a prior on topologies; however, in a Bayesian interpretation, ML is equivalent to maximum a posteriori estimation under a uniform (PDA) prior. PDA produces trees that are highly unbalanced on average, but still under-represents extremely caterpillar-like structures.

Model-based formulations such as the Yule (pure-birth) process (Yule 1925) and the general birth–death process (Kendall 1948) provide well-defined probability distributions over trees and are commonly used to sample random phylogenies. These models tend to generate more balanced tree shapes than PDA, but newer age-dependent generalisations (Andersen et al. 2025) allow interpolation across a broad spectrum of shapes, from highly unbalanced to highly balanced trees. Nonetheless, these processes do not induce uniform sampling over tree space.

Tropical tree space offers new avenues for exploring and sampling phylogenetic tree *distributions*, that is, we want to consider collections of trees, i.e., multiple points in the tropical Grassmannian. For this, we will primarily focus on the specific case of ultrametric trees which, as we shall explain shortly, possess very nice structural characteristics. As before, we use $\mathcal {U}_n\subset \mathcal {T}_n$ to denote the *space of ultrametrics* on n leaves. In the notation of Lemma 3, we have

$$ \mathcal {U}_n \,\cong \, \widetilde{\mathcal {B}}(M(K_n)) \,=\, \operatorname {trop}(M(K_n)), $$

where $\widetilde{\mathcal {B}}$ is called the *Bergman fan* of the matroid $M(K_n)$, named in recognition of George Bergman’s early work on *logarithmic limit sets* (Bergman 1971). The Bergman fan of any matroid (not necessarily $M(K_n)$) *is* the tropical linear space associated with its underlying matroid.

A common question is how to define a measure of closeness between two trees. As discussed in the introduction, BHV distance is one of the most widely used approaches where the distance is both mathematically sound and biologically meaningful; however, its computation is costly and does not always scale well to large datasets (Owen and Provan 2011). The Robinson–Foulds (RF) distance (Robinson and Foulds Feb. 1981), by contrast, is computationally cheap and widely used in practice, but it only captures topological differences (the presence or absence of splits) and ignores branch lengths, which limits its sensitivity. RF distance also saturates quickly and trees can attain a maximal normalised distance of one, despite being quite similar. The Subtree-Prune and Regraft (SPR) distance is another alternative that reflects the minimal number of topological rearrangements needed to transform one tree into another, which ties directly to biologically meaningful evolutionary processes; however, it is NP-hard to compute exactly and often requires heuristics, reducing its practicality for very large trees.

### The Tropical Metric (Generalized Hilbert Projective Metric)

The tropical Grassmannian induces a natural notion of distance between trees via the *tropical metric*. Given two vectors $u, v\in {\mathbb {R}}^n$, the tropical distance is defined as

14 $$\begin{aligned} d_{\textrm{tr}}(u,v) \,{:}{=}\, \max _{i}\,(u_{i}-v_{i}) \,-\, \min _{i}\,(u_{i}-v_{i}). \end{aligned}$$

Intuitively, this distance measures the range of coordinate-wise differences between u and v. It is also easy to see that it is invariant under adding a constant vector to either vector, which we should expect since u and $u+{\zeta }\textbf{1}$ represent the same point in ${{\,\textrm{tropGr}\,}}(2,n)$. Note, however, that $d_{\textrm{tr}}$ is *not* invariant by adding *any* vector from $L_n$, the lineality space of ${{\,\textrm{tropGr}\,}}(2,n)$. In other words, perturbing u or v by an arbitrary element of $L_n$ can, in general, change $d_{\textrm{tr}}(u,v)$.

Tropical distance respects the combinatorial structure of tree space: small topological changes correspond to small tropical distances. From a biological perspective, the tropical distance between two ultrametric trees has a natural interpretation. If two trees, $T_1,\,T_2$, are represented by their distance vectors, ${\mathrm d}^{(1)},\,{\mathrm d}^{(2)}$, where entry ${\mathrm d}^{(\cdot )}_{ij}$ records the divergence time of taxa *i* and *j*, then the coordinate-wise differences ${\mathrm d}^{(1)}_{ij} - {\mathrm d}^{(2)}_{ij}$ measure how much earlier or later each pair of taxa coalesces in one tree relative to the other. Thus this captures the *spread* of these discrepancies: it is the gap between the clade whose divergence is shifted the earliest and the clade whose divergence is shifted the latest across the two trees. Because this metric is invariant under adding a constant to all entries, it ignores uniform shifts in divergence times (e.g. due to calibration), and instead reflects the relative rescaling of divergence events across lineages. In this sense, the tropical distance quantifies the worst-case disagreement in relative evolutionary timing, emphasizing how unevenly the two trees stretch or compress different parts of their histories.

Tropical Line Segments Between Ultrametric Trees Consider two ultrametrics u,v that are both finite, i.e., $u,\,v\in \left\{ \mathcal {U}_n\cap \mathbb {R}^{m}/\mathbb {R}\textbf{1} \right\}$ (recall that $$m=\left( {\begin{array}{c}n\\ 2\end{array}}\right) $$). The *tropical line segment* between $u, v\in \mathbb {R}^{m}/\mathbb {R}\textbf{1}$ is defined as

15 $$\begin{aligned} \Gamma _{u,v} \,=\, \left\{ \ell \odot u\oplus v\,\big |\, \ell \in [\,\min (v-u), \max (v-u)\,] \right\} . \end{aligned}$$

Tropical line segments exist for any two points in $\mathbb {R}^{m}/\mathbb {R}\textbf{1}$, which includes the finite elements of ${{\,\textrm{tropGr}\,}}(2,n)$ (i.e., distance vectors of trees with finite pendant edge lengths). However, for ultrametrics (and by extension for distance vectors of equidistant trees), they acquire particularly nice properties. For starters, note that for any choice of ℓ the resulting point in $\mathbb {R}^{m}/\mathbb {R}\textbf{1}$ remains an ultrametric. That is $\Gamma _{u,v}\subset \mathcal {U}_n$. Thus, by interpolating among ultrametric trees, we are guaranteed to remain in (ultrametric) tree space. The reasons for this stems from the fact that $\mathcal {U}_n$ is a *tropical linear space* and is therefore *tropically convex*, see (Speyer 2008; Lin et al. 2017). Thus, our tropical line segment (anchored by points in a tropically convex set) is simply the tropical analogue of what we are used to when dealing with classical convexity.

**Fig. 3:**
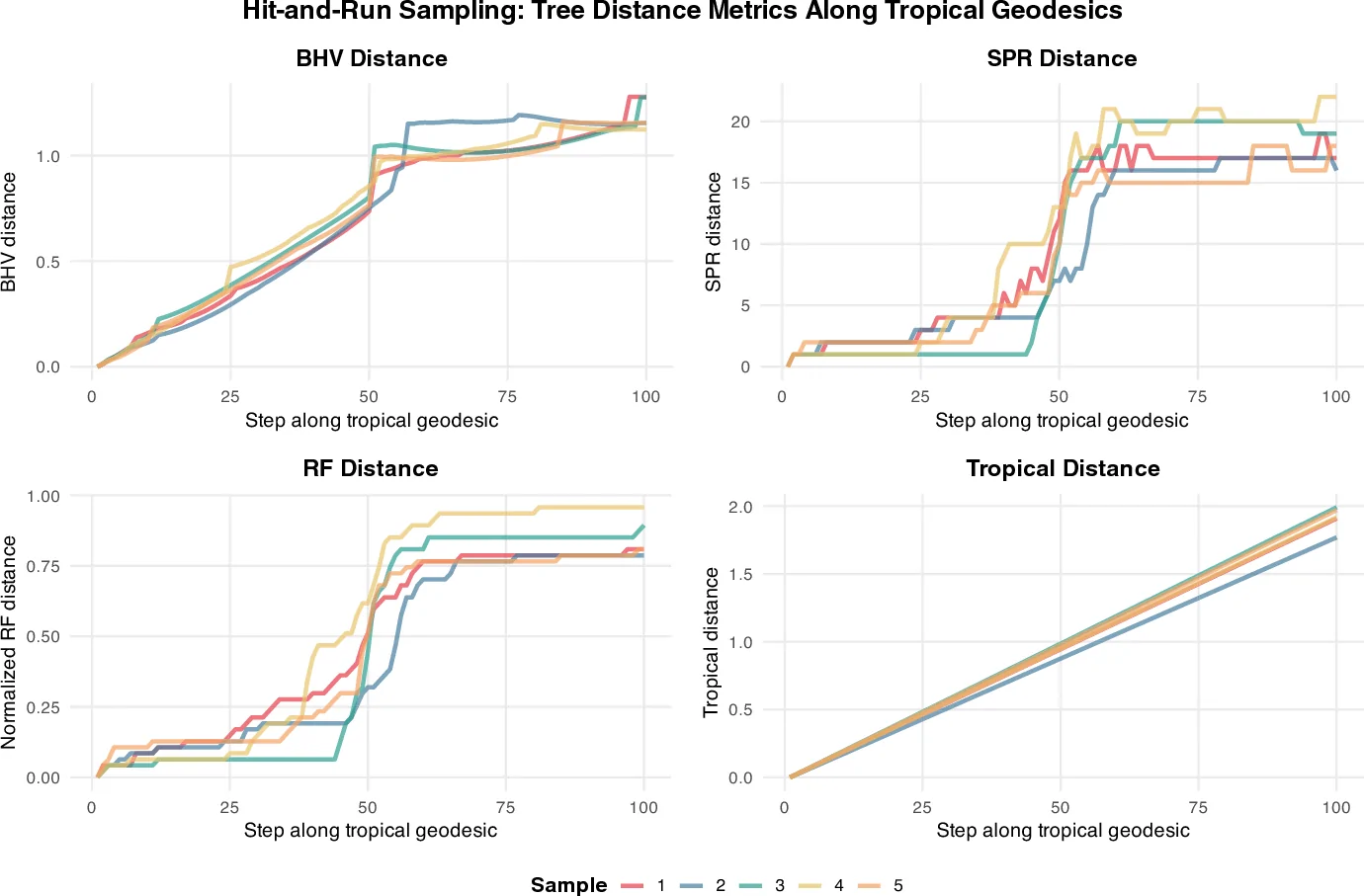
Tree distance metrics along tropical geodesics for five independent hit-and-run samples. Each colored curve corresponds to a separate pair of random coalescent trees connected by a tropical geodesic of 100 interpolation steps. The four panels display the evolution of distances from the starting tree to the interpolated tree as measured by: Billera–Holmes–Vogtmann (BHV) distance (top left), subtree prune-and-regraft (SPR) distance (top right), Robinson-Foulds (RF) distance (bottom left), and tropical distance (bottom right). The consistent qualitative behavior across samples illustrates the regularity of the tropical interpolation, while the variation in magnitude reflects the diversity of tree space geometry (Color figure online)

The interval $[\min (v-u), \, \max (v-u)]$ is exactly the set of scalar shifts ℓ such that the coordinates of $\ell \odot u\oplus v$ interpolate between u and v in the tropical sense. Hence, the tropical line segment $\Gamma _{u,v}$ is parameterized by ℓ in this interval. From this it becomes simple to sample trees between two anchor trees using Eq. (15). We see from Fig. 3 that we can sample two ultrametric trees from a coalescent process and adjusting ℓ in Eq. (15) allows us to smoothly interpolate between these two trees. As expected, the tropical distance is perfectly linear between these two trees across the line segment, but this distance is closely correlated with BHV, SPR and RF distances too. For details on how tree topologies change as one interpolates along the tropical line segment, see (Yoshida and Cox 2022).

In Fig. 3 we demonstrate this *smoothness* by constructing tropical line segments between five independent pairs of utrametric trees and tracking four alternative distances (BHV, SPR, RF, and tropical). We (i) verify that interpolation by $\ell \odot u\oplus v$ remains within the ultrametric tree space, and (ii) observe that the tropical distance varies *exactly linearly* along the path reflecting its tropical projective invariance. By contrast, BHV distances change continuously but with variable rate (as seen by the non-uniform slopes), and SPR/RF update in discrete steps.

The Tropical Convex Hull of a Set of Ultrametric Trees Since tree metrics are implicitly finite, our decision to operate with the tropical projective torus, $\mathbb {R}^{m}/\mathbb {R}\textbf{1}$, as our ambient space is perfectly reasonable. More generally, however, one could consider *tropical projective space*, $\mathbb{T}\mathbb{P}^{m-1}$, defined as

$$ \mathbb{T}\mathbb{P}^{m-1}{:}{=}\bigl (\mathbb {T}_{\max }^m {\setminus } \{(-\infty ,\dots ,-\infty )\}\bigr ) / {\mathbb {R}}\textbf{1}. $$

$\mathbb{T}\mathbb{P}^{m-1}$ is the tropical analogue of classical projective space, and is obtained in a similar fashion. That is, we identify vectors in $\mathbb {T}_{\max }^n$ that differ by (tropical) scalar multiplication, removing the “zero” vector prior to quotienting. From the definition, we see that $\mathbb {R}^{m}/\mathbb {R}\textbf{1}$ is just the finite subset of $\mathbb{T}\mathbb{P}^{m-1}$. It is in this larger space, however, that Arilda’s equivalence between $\widetilde{\mathcal {B}}(M(K_n))$ and $\mathcal {U}_n$ is formulated (Ardila 2005). In fact, Ardila showed that the extremal generators (vertices) of $\mathcal {U}_n$ lie on the boundary strata, meaning they have at least one coordinate equal to -∞ and therefore lie outside $\mathbb {R}^{m}/\mathbb {R}\textbf{1}$. The *tropical convex hull* (defined below) of these (non-finite) generators is precisely $\widetilde{\mathcal {B}}(M(K_n))$. These vertices of $\mathcal {U}_n$ can be computed directly from $\operatorname {trop}(M(K_n))$ as the image under the valuation map of the maximal proper *flats* of $M(K_n)$. By enumerating all such maximal flats, it is (in theory^6^) possible to make explicit the tropical polytope (tropical convex hull) of $\mathcal {U}_n$.

Given a finite set $\mathcal {S}=\{u^{(1)},\dots ,u^{(k)}\} \subset \mathbb{T}\mathbb{P}^{m-1}$, the tropical convex hull, $\operatorname {tconv}$, is the smallest tropically convex subset of $\mathbb{T}\mathbb{P}^{m-1}$ containing $\mathcal {S}$. Formally,

16 $$\begin{aligned} \operatorname {tconv}\bigl (u^{(1)},\dots ,u^{(k)}\bigr ) \,{:}{=}\, \Bigl \{ \,\bigoplus _{j=1}^{k} \lambda _{j} \odot u^{(j)} \,\Big |\, \lambda _{1},\dots ,\lambda _{k} \in \mathbb {T}_{\max }\,\Bigr \}. \end{aligned}$$

Like their classical counterparts, tropical convex hulls generalizes (tropical) line segments to higher dimensions, and they form *tropical polytopes* in their respective ambient space. Thus, $\widetilde{\mathcal {B}}(M(K_n))$ is a tropical polytope in $\mathbb{T}\mathbb{P}^{m-1}$.

Just as classical polytopes are (classically) convex, tropical polytopes are tropically convex, and it is this feature that allows us to naturally extend the closure we observed when taking tropical line segments between pairs of ultrametrics to sets of ultrametrics. Thus we can perform such “interpolation” on $\mathcal {S}\subset \mathcal {U}_n$ without ever leaving (ultrametric) tree space. Namely, any point $w \in \operatorname {tconv}(u^{(1)},\dots ,u^{(k)})$ has coordinates

$$ w_{i} \,=\, \max _{j=1,\dots ,k}\,\bigl (u^{(j)}_{i}+\lambda _{j}\bigr ), \qquad i=1,\dots ,\genfrac(){0.0pt}1{n}{2}. $$

Sampling within $\operatorname {tconv}(\mathcal {S})$, $\mathcal {S}=(u^{(1)},\dots ,u^{(k)})\subset \mathcal {U}_n\cap \mathbb {R}^{m}/\mathbb {R}\textbf{1}$, thus provides a way to interpolate among several anchor trees at once, always producing valid ultrametric trees from within $\mathcal {U}_n$.

Tropical Projection Given a finite set $\mathcal {S}=(u^{(1)},\dots ,u^{(k)})\subset \mathcal {U}_n$ of ultrametrics, suppose we have $w\notin \mathcal {U}$ and want to find the closest (in the sense of the tropical metric) ultrametric to *w* that is still contained in $\operatorname {tconv}(\mathcal {S})$. We use $\mathcal {P}$ to denote the tropical polytope, i.e. $\mathcal {P}=\operatorname {tconv}(\mathcal {S})$. The *tropical projection* of $w \in \mathbb{T}\mathbb{P}^{m-1}$ onto $\mathcal {P}$ is

17 $$\begin{aligned} \pi _{\mathcal {P}}(w) \,=\, \bigoplus _{i=1}^{k} \lambda _i \odot u^{(i)}, \qquad \lambda _i \,=\, \max \{\lambda \in \mathbb {R}\mid \lambda \odot u^{(i)} \le w\} \,=\, \min _{j}\bigl (w_j - u^{(i)}_j\bigr ). \end{aligned}$$

Note that the formula above is defined for general $\mathcal {S}\subset \mathbb{T}\mathbb{P}^{m-1}$ and $w\in \mathbb{T}\mathbb{P}^{m-1}$, not just for finite ultrametrics. However, in the particular case, when $w\in \mathbb {R}^{m}/\mathbb {R}\textbf{1}$ is finite, and $\mathcal {P}=\widetilde{\mathcal {B}}(M(K_n))$ is the Bergman Fan of ultrametric treespace, tropical projection onto $\mathcal {P}$ is known to coincide the well-known (and polynomial time) single-linkage hierarchical clustering algorithm (Ardila 2005) (up to scalar translation). This correspondence is important specifically because it avoids the need to explicitly enumerate all the generators of $\widetilde{\mathcal {B}}(M(K_n))$, which grows exponentially with the size of n. Thus, we leverage the single-linkage algorithm heavily in our approach to sampling $\mathcal {U}_n$.

### Tropical Hit and Run over $\mathcal {U}_n$

Smooth interpolation along tropical line segments anchored by ultrametric trees enables the use of Markov Chain Monte Carlo techniques by sampling along tropical line segments as a subroutine. Such a scheme was proposed in Yoshida et al. (2023) under the name *tropical hit and run* (HAR), where it was applied to both arbitrary tropical polytopes and the space of ultrametrics. The essence of the approach is as follows. Given a tropical polytope $\mathcal {P}=\operatorname {tconv}(\mathcal {S})$ and an initial point $x\in \mathcal {P}$, sample another point $y\in \mathcal {P}$ via some predetermined method. With *x* and *y*, a point *z* is sampled uniformly from the tropical line segment $\Gamma _{x,y}$ as the next proposed move. The algorithm then either accepts *z*, setting x←z, or rejects it, keeping *x* unchanged. The procedure then repeats until termination. The output is a subset of accepted moves encountered throughout the course of the algorithm.

The primary challenge in implementing tropical HAR is in the selection of *y*. For arbitrary $\mathcal {P}$ the authors of Yoshida et al. (2023) propose sampling from among the tropical vertices, or alternatively, sampling from some Euclidean space followed by tropically projecting *y* onto $\mathcal {P}$. As we have seen, the former method requires explicit representation of the vertices of $\mathcal {P}$, which can be prohibitive for trees with many leaves. In particular, it requires enumerating the cocircuits^7^ of $M(K_n)$, of which there are $2^{(n-1)}-1$. Furthermore, tropical line segments with one endpoint given by a vertex of $\mathcal {P}$ tends to result in a considerable portion of the samples coming from cones of the $\widetilde{\mathcal {B}}(M(K_n))$ that are not of maximum possible dimension, i.e. ultrametrics corresponding to non-binary (unresolved) topologies.

The latter method (sampling from some Euclidean space followed by tropically projecting *y* onto $\mathcal {P}$), also requires explicit vertex representation in the general case. Fortunately, as discussed earlier, for $\mathcal {P}=\widetilde{\mathcal {B}}(M(K_n))$, we can leverage the single-linkage, effectively bypassing this requirement. Even so, we must proceed with some care, as distributions associated with sampling over a Euclidean space do not translate once tropical projection is applied because such a mapping is not injective. This limitation notwithstanding, our overall procedure is outlined in Algorithm 3, where we use $\mathcal {P}$ to denote the tropical polytope corresponding to $\mathcal {U}_n$.

**Figure:**
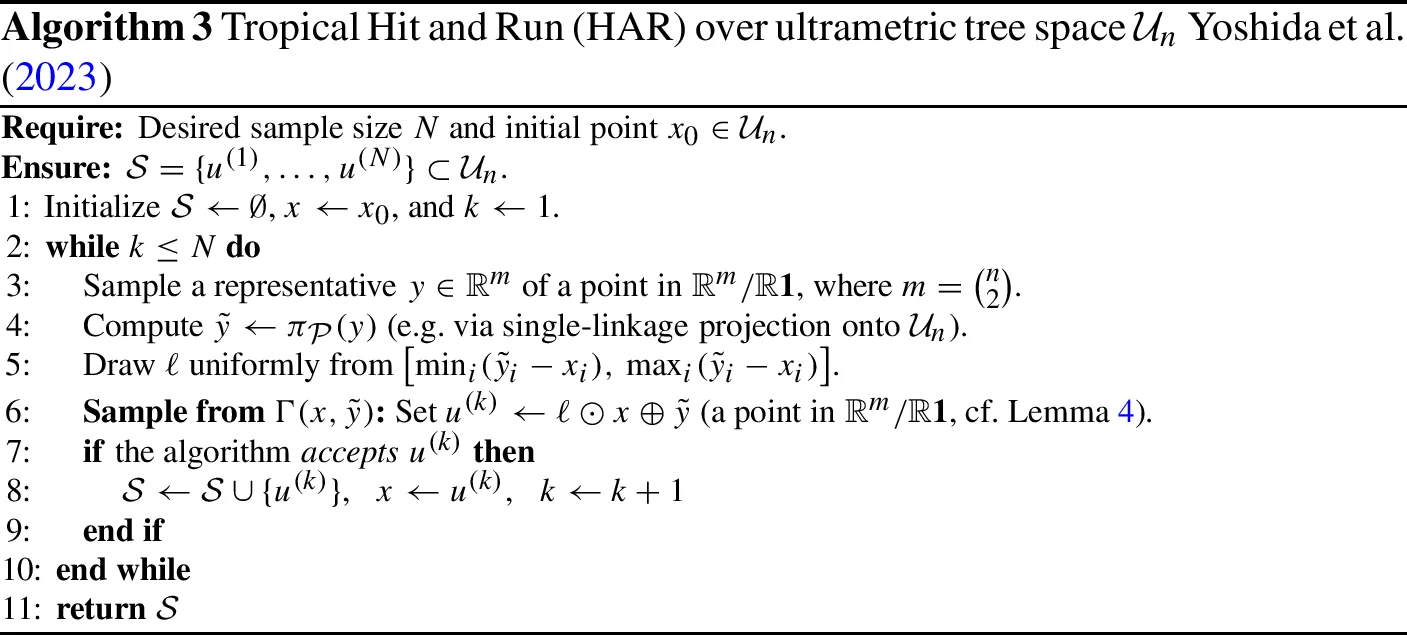

Since points in $\mathbb {R}^{m}/\mathbb {R}\textbf{1}$ are defined only up to adding a constant multiple of $\textbf{1}$, Algorithm 3 should not depend on the particular representatives chosen for x and $\tilde{y}$. The next lemma verifies this invariance for the tropical line-segment proposal, ensuring that the hit and run step is intrinsically defined on the tropical projective torus.

#### Lemma 4

(Well-definedness of Algorithm 3 on $\mathbb {R}^{n}/\mathbb {R}\textbf{1}$) Let $$m= \left( {\begin{array}{c}n\\ 2\end{array}}\right) $$ and let $\mathbb {R}^{m}/\mathbb {R}\textbf{1}$ denote the tropical projective torus. In Algorithm 3, fix points $x, \tilde{y} \in \mathbb {R}^{m}/\mathbb {R}\textbf{1}$ and choose representatives $x,\tilde{y} \in {\mathbb {R}}^{m}$. Define

$$ u:= \ell \odot x \oplus \tilde{y} \;\in \; {\mathbb {R}}^{m} \qquad \text {where}\qquad \ell \in \bigl [\min _i(\tilde{y}_i-x_i),\,\max _i(\tilde{y}_i-x_i)\bigr ]. $$

Then the equivalence class of *u* in $\mathbb {R}^{n}/\mathbb {R}\textbf{1}$ is independent of the choice of representatives for *x* and $\tilde{y}$. In particular, the proposal $u^{(k)} = \ell \odot x \oplus \tilde{y}$ in Algorithm 3 is well-defined as a point of $\mathbb {R}^{m}/\mathbb {R}\textbf{1}$.

#### Proof

Let $x',\tilde{y}' \in {\mathbb {R}}^{m}$ be another choice of representatives for the same points in $\mathbb{T}\mathbb{P}^{m-1}$. Then there exists $\alpha \in {\mathbb {R}}$ such that $x' = x + \alpha \textbf{1}$ and $\tilde{y}' = \tilde{y} + \alpha \textbf{1}$. For each coordinate *i* we have

$$ \tilde{y}'_i - x'_i = (\tilde{y}_i + \alpha ) - (x_i + \alpha ) = \tilde{y}_i - x_i, $$

so the interval $\bigl [\min _i(\tilde{y}_i-x_i),\,\max _i(\tilde{y}_i-x_i)\bigr ]$ is the same for $(x,\tilde{y})$ and $(x',\tilde{y}')$. In particular, a given choice of ℓ in this interval is valid for both pairs of representatives. Using max-plus notation, we compute

$$ \ell \odot x' \oplus \tilde{y}' = \max (x'+\ell ,\tilde{y}') = \max (x+\alpha \textbf{1}+\ell ,\tilde{y}+\alpha \textbf{1}) = \max (x+\ell ,\tilde{y}) + \alpha \textbf{1}= \bigl (\ell \odot x \oplus \tilde{y}\bigr ) + \alpha \textbf{1}. $$

Thus the proposal $u^{\prime }$ obtained from $$(x',\tilde{y}')$$ satisfies $$u' = u + \alpha \textbf{1}$$, so *u* and $u^{\prime }$ define the same class in $$\mathbb {R}^{m}/\mathbb {R}\textbf{1}$$. Therefore the point *u* is well-defined on the quotient, and therefore it is independent of the choice of representatives for *x* and $$\tilde{y}$$. □

Lemma 4 ensures that the sampling step in Algorithm 3 is independent of arbitrary normalization choices, see Remark 2.

#### Remark 7

Algorithm 3 can be generalized to sample from any tropical polytope $\mathcal {P}$ if one uses Eq. (17) to compute the tropical projection $\pi _\mathcal {P}(y)$ rather than the single-linkage algorithm. But again, this requires one to have all the vertices of $\mathcal {P}$ explicitly.

**Fig. 4:**
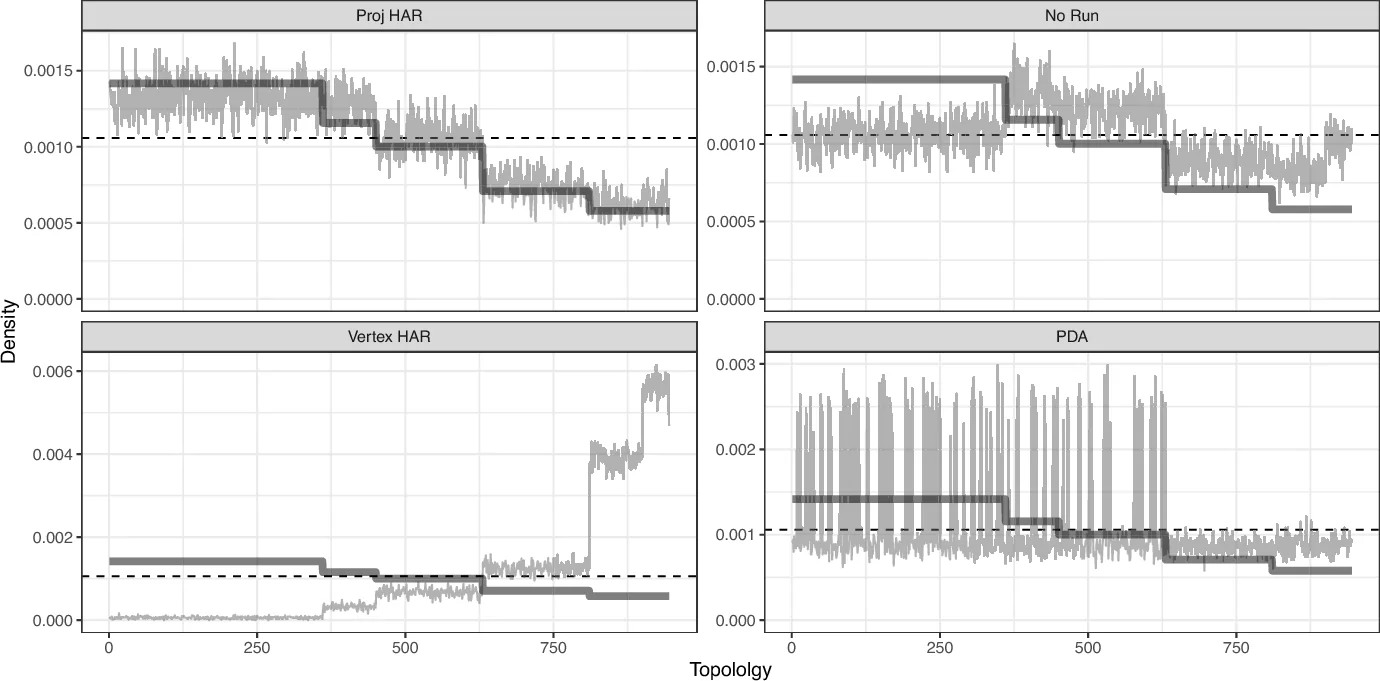
Density plots by tree topology for N=1000 samples obtained using variations of the tropical HAR algorithm. The projective HAR method (top-left) utilizes Algorithm 3. The “No Run” method (top-right) also uses Algorithm 3, but skips lines 5-6 and sets $u^{(k)}\leftarrow \tilde{y}$ directly. Vertex HAR (bottom-left) modifies Algorithm 3 in line 3 by sampling *y* from (the vertices of) $\mathcal {P}$ directly. PDA (bottom-right) modifies line 3 by computing *y* using the R package TreeTools. The piecewise-linear lines represent the normalized cone volumes for each corresponding topology

We now provide a small demonstration of the preceding sampling method. Using Algorithm 3, we sample N=1,000 ultrametric from $\mathcal {U}_6$, and record the topology^8^ of each collected tree metric. We sampled *y* uniformly from the unit hypercube. For comparative purposes, we also include the ultrametrics obtained by excluding the “run” step, i.e., simply setting $u^{(k)}\leftarrow \tilde{y}$ in each iteration without sampling from the tropical line segment. Intuitively, we expect there to be a bias associated with “no run” sampling that is proportional to the *size*^9^ of the topology’s corresponding cone in $\tilde{\mathcal {B}}(M(K_n))$. A reasonable expectation might be that “larger” topologies (in the sense of their cones’ size) will be sampled more frequently. We include a normalized measure of each cone’s volume in our results and sort the topologies according to their relative sizes (largest to smallest). The results of this comparison are shown in Fig. 4.

**Fig. 5:**
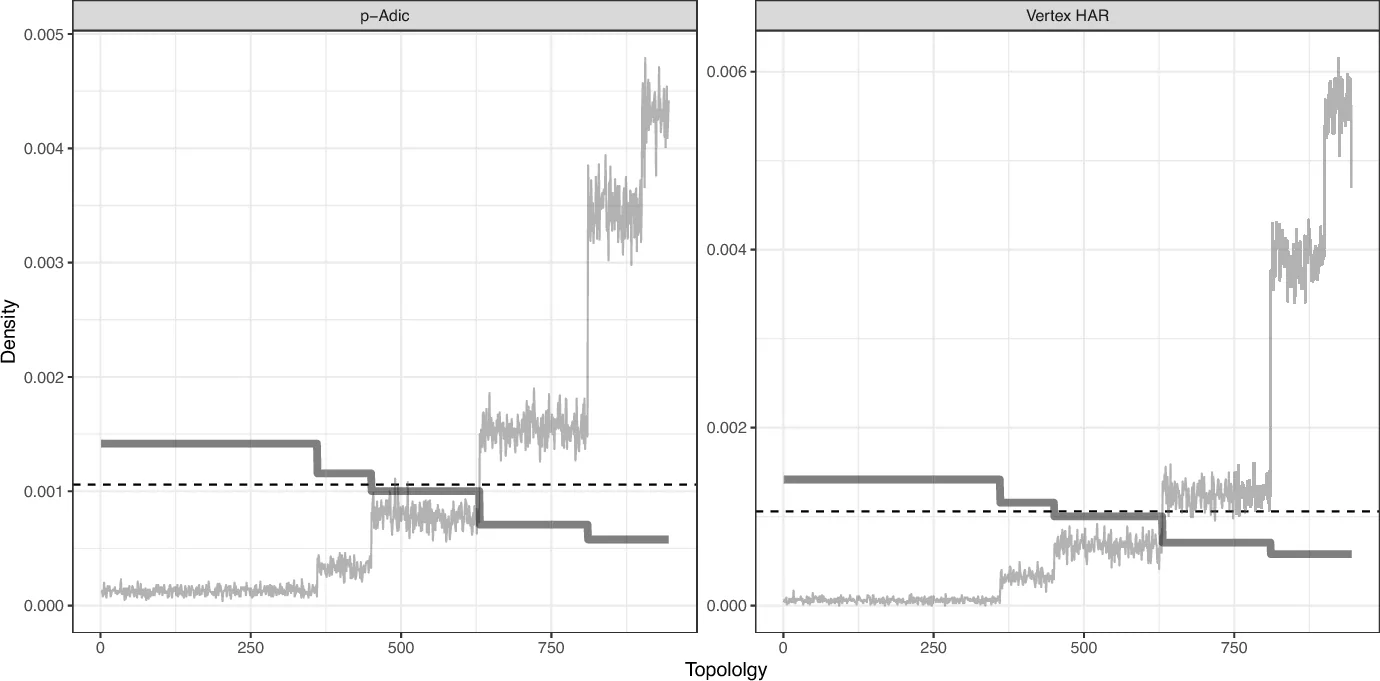
Density plots by tree topology for N=1000 samples obtained using 2-adic sampling via Algorithm 2 (left) and Hit & Run using the vertices of $\tilde{\mathcal {B}}(M(K_6))$ (Vertex HAR). Similar trends in sampling frequencies between these methods should be explored for larger values of *p* and *n*

It is interesting to note that HAR’s sampling frequency is much more closely aligned with volume than that of the “no run” method, though both methods over- and under-sample considerably across the range of topologies. We also include the vertex HAR method, which selects *y* from amongst the vertices of $\widetilde{\mathcal {B}}(K_6)$ at each step, and the PDA method^10^ from the TreeTools package (Smith 2019) in R. We also sampled (non-equidistant trees) using Algorithm 2 with p=2. By extending pendant edges as necessary to obtain an equidistant tree, we were able to compare the sampling frequency of each topology as before. Interestingly, the resulting topologies followed a frequency pattern very similar to that of the Vertex HAR method, as shown in Fig. 5. Extending such computational experiments to other values of *p* and to larger number of leaves constitutes an opportunity for follow-on research.

**Fig. 6:**
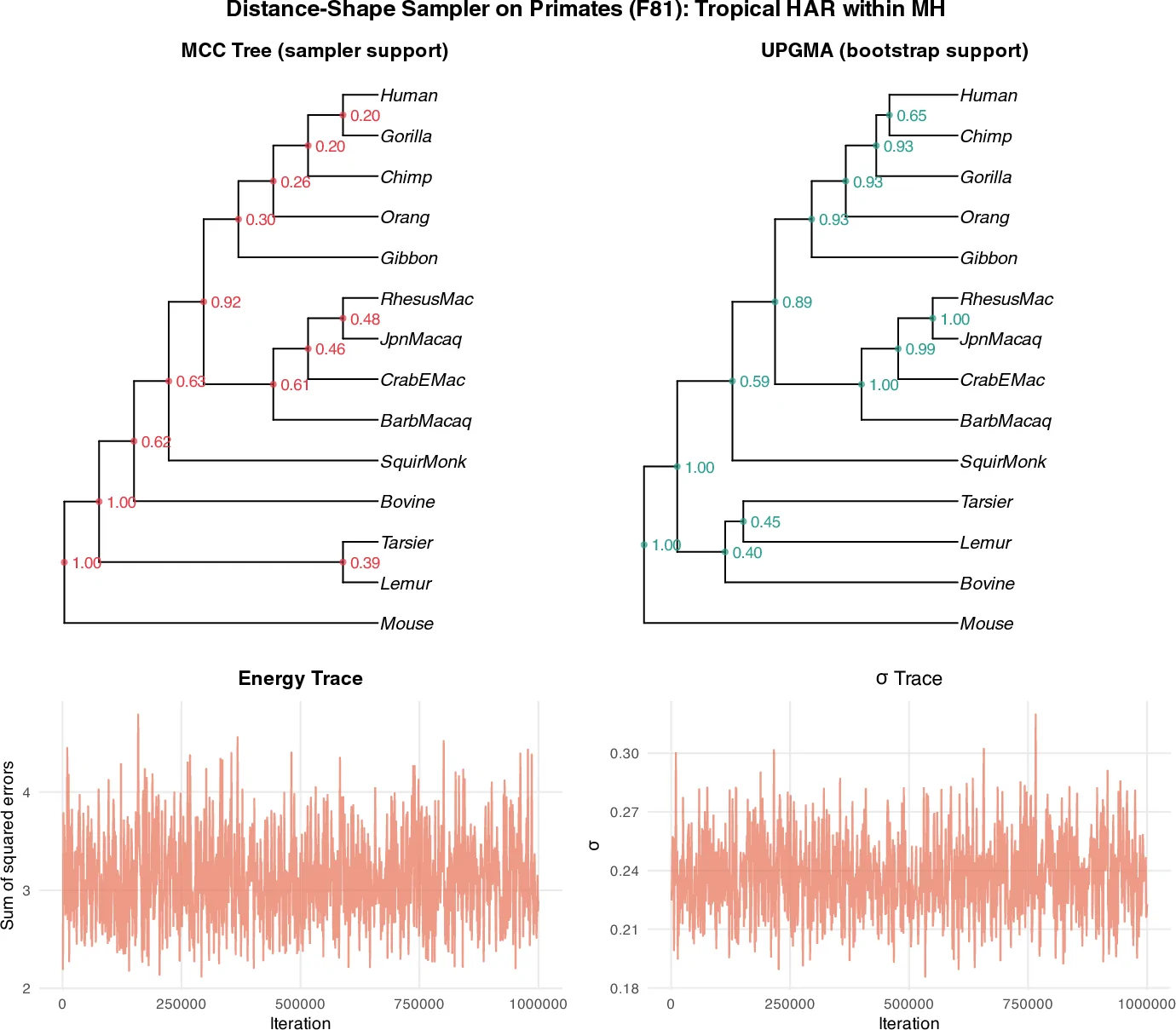
Distance-shape sampler on the primates alignment (12 taxa, F81). **Top left:** MCC tree with sampler clade support (red). **Top right:** UPGMA tree with bootstrap support (teal, 1000 replicates). **Middle:** energy and σ traces. **Bottom:** BHV and Robinson–Foulds distances from the starting tree. The sampler recovers the same topology as bootstrap UPGMA with comparable support values (Color figure online)

## Illustrative Example: Distance-Shape Sampling

In this final section, we apply the tropical Hit-and-Run sampler from Algorithm 3 to a phylogenetic inference problem on the primates nucleotide alignment (n=12 taxa) provided by the R package phangorn (Schliep 2011). We compute an empirical pairwise distance matrix $D^{\textrm{emp}}$ under the F81 model and extract its lower-triangular vector $d^{\textrm{emp}}\in \mathbb {R}^m$. Each candidate tree *T* is represented by its ultrametric distance vector $d(T)\in \mathbb {R}^m$.

Since distance vectors are meaningful only up to global scale (cf. the lineality space $L_n$), we compare a tree to the data after proportional alignment: $A(d(T); d^{\textrm{emp}}) = \hat{\alpha }\, d(T)$, where $\hat{\alpha } > 0$ minimises $\sum _k (d_k^{\textrm{emp}} - \alpha \, d_k(T))^2$. The energy $E(T) = \sum _k (d_k^{\textrm{emp}} - \hat{\alpha }\, d_k(T))^2$ measures mismatch in distance shape after removing the rate ambiguity. We define the target

$$ \pi (T, \sigma ^2) \propto (\sigma ^2)^{-m/2}\exp \!\left( -\frac{E(T)}{2\sigma ^2}\right) \textrm{InverseGamma}(\sigma ^2; a_0, b_0), $$

where $\sigma ^2$ acts as an adaptive temperature. The sampler alternates two steps:

1. **Temperature update** Draw $\sigma ^2 \mid T \sim \textrm{InvGamma}(a_0 + m/2,\; b_0 + E(T)/2)$.
2. **Tree update** Let $x {:}{=}\vert \vert d(T)\vert \vert _{\textrm{tr}}$ denote the tropical norm of the distance vector. We generate a local direction *y* by perturbing *x* with Gaussian noise and mapping the result to an ultrametric tree via single-linkage UPGMA, with the perturbation scale adaptively tuned to achieve an acceptance rate of approximately 0.28. We then sample a point *z* on the tropical line segment between *x* and *y* (Algorithm 3), and map *z* to an ultrametric tree $T^\star$ again using single-linkage UPGMA. The proposal is accepted with probability
  $$ p_{\textrm{acc}}(T, T^\star ) = \min \!\left\{ 1,\; \exp \!\left( -\frac{E(T^\star ) - E(T)}{2\sigma ^2}\right) \right\} . $$

For this illustrative example, the sampler highlights one practical use case of the tropical HAR proposal while keeping the implementation relatively simple. The only nonlinear projection occurs in the construction of the proposal direction y, where a perturbed distance vector is mapped to an ultrametric tree by UPGMA. Once both the current state x and the endpoint y lie in the ultrametric cone, the tropical line segment between them remains ultrametric by tropical convexity of the Bergman fan. Thus, no additional projection is needed after the tropical line step. However, because the UPGMA-based construction of y induces an implicit and generally intractable proposal density, the corresponding Hastings correction cannot be evaluated. Consequently, the resulting accept–reject procedure should be interpreted as a heuristic Metropolis-type sampler rather than an exact Metropolis–Hastings algorithm.

The resulting distribution is shown in Fig. 6, alongside a bootstrap UPGMA tree constructed from 1000 nonparametric replicates of the alignment. The sampler recovers a biologically sensible tree whose topology closely matches the bootstrap UPGMA. Clade support values are broadly comparable but somewhat more diffuse, likely because the sampler sees only a single fixed distance matrix rather than resampled alignment columns, and therefore cannot distinguish topologies that produce similar pairwise distances. Minor topological differences between the MCC tree and the UPGMA tree are expected, since the two methods optimise different objectives: the sampler minimises a scaled least-squares fit with adaptive temperature, whereas UPGMA constructs a single tree by greedy average-linkage clustering. This example demonstrates that the tropical Hit-and-Run machinery developed in earlier sections can be embedded in a practical inference loop, providing a geometrically motivated approach to exploring ultrametric tree space with uncertainty quantification.

## Conclusions

The tropical Grassmannian offers a unifying, algebraically rigorous framework for understanding tree space. By tropicalizing the Plücker relations, we recover the four-point condition and ensure that every point of ${{\,\textrm{tropGr}\,}}(2,n)$ corresponds to a valid tree metric. This reveals that the vast combinatorial complexity of phylogenetic trees can be encoded by relatively few parameters and expressed within the rich structure of tropical geometry. Moreover, the tropical viewpoint connects naturally to ultrametrics, Bergman fans, and novel distance measures such as the tropical metric, each of which provides fresh perspectives on phylogenetic inference.

At the same time, our exploration highlights practical challenges. Sampling from ${{\,\textrm{tropGr}\,}}(2,n)$ via uniform draws in ${{\,\textrm{Gr}\,}}^{\circ }(2,n)$ produces highly unbalanced trees, distinct from biologically realistic models such as Yule or coalescent processes. Likewise, while the tropical metric is computationally simple and respects combinatorial tree structure, its biological interpretation remains less clear compared to BHV, SPR, or RF distances.

Future work should focus on developing biologically meaningful sampling strategies in tropical space, connecting tropical geometry with stochastic models of evolution, and exploring the use of tropical optimization techniques in inference. In doing so, the tropical Grassmannian has the potential not only to deepen our theoretical understanding of tree space, but also to provide new algorithmic tools for phylogenetic reconstruction in large and complex datasets.

## Footnotes

1 Cartan–Alexandrov–Toponogov.

2 Any unrooted tree can be made rooted by adding an internal node on the relative interior of any internal edge.

3 If $\textrm{lca}(i,j,k)$ is not trivalent with respect to $\{i,j,k\}$ then this partition is not unique and any choice of the singleton and corresponding pair is valid.

4 For the reader who is curious about matroids the authors recommend (Gordon and Mcnulty 2012), which provides a delightful introduction to the topic. See also (Oxley 2006) for more rigorous treatment.

5 Another way to arrive at this conclusion is to consider the special case of ultrametrics. For any ultrametric vector *u* it is easy to see that $u+{\zeta }\textbf{1}$ for any ${\zeta }\in {\mathbb {R}}$ is also an ultrametric and that this does not hold for any other vector except $\textbf{1}$. Thus $\textbf{1}$ *defines* the lineality space for the space of ultrametric trees. Since ultrametric tree space is contained in tree space, $\textbf{1}$ must be an element of ${{\,\textrm{lin}\,}}\left( {{\,\textrm{tropGr}\,}}(2,n)\right)$.

6 We say “in theory” because any algorithm for enumerating flats for $M(K_n)$ has a worst-case running time that is exponential in the input size (Oxley 2006), and so this quickly becomes infeasible for trees with many leaves.

7 Briefly, every matroid *M* has a dual matroid $M^*$ over the same ground set. Circuits of *M* correspond to *cocircuits* of $M^*$ and vice versa. We use cocircuits in our earlier statement, as these are in fact the complement of (and therefore combinatorially equivalent to) the maximal flats of *M*.

8 By “topology” of ultrametric *u*, we mean the index of the minimal closed cone of $\widetilde{\mathcal {B}}(M(K_6))$ containing *u*. Our *acceptance* criteria in each case is that the sample has a fully-resolved topology, meaning that $u^{(k)}$ is contained in the relative interior of a maximal cone of $\widetilde{\mathcal {B}}(M(K_n))$. For n=6 (a small number of leaves), full enumeration of the generators (vertices) of $\widetilde{\mathcal {B}}(M(K_n))$ is manageable. As each cone is defined by a subset of these generators, checking containment can be performed as a linear programming feasibility problem. Other (faster) methods also exist.

9 There are different notions of *size* one can use. Here, we use the Euclidean volume of the cones’ intersection with the unit hypercube. This is also why we sample *y* from a hypercube as opposed to, say, the unit hypersphere - the intersections are easier to compute in our case.

10 By default, the RandomTrees function of TreeTools does not produce ultrametrics, however, such trees can be made into ultrametrics without changing the underlying topology by extending pendant edges as required.
